# Targeted Ablation of Nesprin 1 and Nesprin 2 from Murine Myocardium Results in Cardiomyopathy, Altered Nuclear Morphology and Inhibition of the Biomechanical Gene Response

**DOI:** 10.1371/journal.pgen.1004114

**Published:** 2014-02-20

**Authors:** Indroneal Banerjee, Jianlin Zhang, Thomas Moore-Morris, Emily Pfeiffer, Kyle S. Buchholz, Ao Liu, Kunfu Ouyang, Matthew J. Stroud, Larry Gerace, Sylvia M. Evans, Andrew McCulloch, Ju Chen

**Affiliations:** 1 Department of Medicine, University of California-San Diego, La Jolla, California, United States of America; 2 Skaggs School of Pharmacy and Pharmaceutical Sciences, University of California-San Diego, La Jolla, California, United States of America; 3 Department of Bioengineering, University of California-San Diego, La Jolla, California, United States of America; 4 Drug Discovery Center, Key Laboratory of Chemical Genomics, Peking University Shenzhen Graduate School, Shenzhen, China; 5 Department of Cell and Molecular Biology, The Scripps Research Institute, La Jolla, California, United States of America; Children's Hospital Boston and Harvard Medical School, United States of America

## Abstract

Recent interest has focused on the importance of the nucleus and associated nucleoskeleton in regulating changes in cardiac gene expression in response to biomechanical load. Mutations in genes encoding proteins of the inner nuclear membrane and nucleoskeleton, which cause cardiomyopathy, also disrupt expression of a biomechanically responsive gene program. Furthermore, mutations in the outer nuclear membrane protein Nesprin 1 and 2 have been implicated in cardiomyopathy. Here, we identify for the first time a role for the outer nuclear membrane proteins, Nesprin 1 and Nesprin 2, in regulating gene expression in response to biomechanical load. Ablation of both Nesprin 1 and 2 in cardiomyocytes, but neither alone, resulted in early onset cardiomyopathy. Mutant cardiomyocytes exhibited altered nuclear positioning, shape, and chromatin positioning. Loss of Nesprin 1 or 2, or both, led to impairment of gene expression changes in response to biomechanical stimuli. These data suggest a model whereby biomechanical signals are communicated from proteins of the outer nuclear membrane, to the inner nuclear membrane and nucleoskeleton, to result in changes in gene expression required for adaptation of the cardiomyocyte to changes in biomechanical load, and give insights into etiologies underlying cardiomyopathy consequent to mutations in Nesprin 1 and 2.

## Author Summary

Cardiomyopathy is one of the major causes of heart failure, and the leading cause of mortality in the western world. A number of cardiomyopathies are caused by genetic mutations in structural proteins within the cardiomyocyte. Recent interest has turned to structural proteins that link the nucleoskeleton to the cytoskeleton (The LINC complex). Disruption of these factors cause both mechanical and gene response changes that cause varying degrees of pathology. In particular two genes, Nesprins 1 and 2 are of key interest as they share a high degree of homology and both have been found in Emery-Dreifuss muscular dystrophy (EDMD) patients with cardiomyopathy. Studies of these factors in the heart; however, have been limited due to early lethality of dual ablation of both Nesprins 1 and 2 in global knockout mice. Here, we examine targeted genetic ablation of Nesprin 1 and/or Nesprin 2 in a novel set of mouse lines. These studies found that loss of these factors caused early onset of cardiomyopathy, changes in nuclear position and morphology, as well as inhibition of the biomechanical gene response. Together, these data demonstrate that Nesprin 1 and 2 play critical and overlapping roles in the heart during homeostasis and pathology.

## Introduction

Mechanical cues from the extracellular matrix (ECM) transmitted through the cytoskeleton to the nucleoskeleton are regulated by a unique set of structural protein complexes [1], [2]. These mechanotransducers function as a platform to translate mechanical cues into biochemical signals and effect a wide range of biological functions (development, migration, cell specification) [2]. Recent attention has turned to protein complexes at the nucleus, their regulation of mechanotransduction and how these structures affect the biomechanical properties of the cell and tissue [1]. Indeed, a number of pathologies, including dilated cardiomyopathy (DCM) and Emery-Dreifuss muscular dystrophy (EDMD), arise from mutations within cardiomyocyte structural proteins affecting mechanical signal integration [3], [4]. Given this, better understanding of structural proteins within the nucleus involved in sensing biomechanical load and enabling adaptive cellular responses during cardiovascular development and pathology can lend significant insight into our understanding of cardiac function [5].

The nucleus itself is separated from the cytoplasm via the nuclear envelope (NE) which is comprised of two membranes; the Outer Nuclear Membrane (ONM) and the Inner Nuclear Membrane (INM) [6]–[8]. Both the ONM and INM contain elements of a protein complex that mechanically link the nucleoskeleton to the cytoskeleton termed the LINC complex (linker of nucleoskeleton and cytoskeleton). The LINC complex is comprised of a number of proteins from two families, the SUN proteins (Sad1p/UNC-84) anchored within the INM, and the Nesprins at the ONM [9]–[13].

The Nesprin family is comprised of four members (Nesprin 1–4) that anchor nuclei to the actin cytoskeleton, intermediate filaments and the microtubule cytoskeleton. Two members, Nesprin 1 and 2, link the nuclear lamina to the actin cytoskeleton and share 65% homology [9], [11], [13]–[16]. Both Nesprin 1 and Nesprin 2 use alternative promoters, RNA splicing and termination to give rise to multiple isoforms, ranging from 61 kDa–1.01 MDa, with the two largest being the giant Nesprins 1G (1.01 MDa) and Nesprin 2G (796 KDa) [9], [12], [17]. Structurally, Nesprin 1G and 2G contain two N-terminal Calponin-homology (CH) domains responsible for linkage to the actin cytoskeleton, a number of Spectrin-repeat (SR) rod domains and a C-terminal KASH (Klarsicht/Anc/Syne Homology) domain [11], [12], [14]–[16], [18]. The KASH domain is key in the interaction of Nesprins with their LINC complex partners, SUN proteins. Recent crystal structures have shown that Nesprin 1 or 2 function in the LINC complex by localizing to the ONM and linking to a homotrimeric SUN domain containing protein complex (either SUN1 or SUN2) via the KASH domain at the INM [19], [20].

In addition to Nesprin and SUN proteins, the INM contains a number of LINC associated factors, including the nuclear lamina, comprised of both A- and B- type Lamins and Emerin. The Lamins are type V intermediate filaments that comprise the protein meshwork that lines the INM and physically associate with the LINC complex via direct interactions with SUN proteins. A-type lamins (Lamin A and C) are alternatively spliced isoforms of the *LMNA* gene, whereas B-type lamins (Lamin B1 and B2) are encoded by two separate genes *LMNB1* and *LMNB2* [21]. Emerin, encoded by *EMD*, is an integral INM protein that can interact with both SUN and Lamin A/C and affects mechanical load responses in cells [22]. Both of these LINC associated factors were found at the cellular level to be critical for mechanotransduction, cellular stiffness, nuclear positioning and nuclear envelope integrity in a number of cell types [21]–[25]. These data suggest that other factors in the nucleus could also affect mechanical loading and nuclear structure/positioning.

Mutations in both *LMNA* and *EMD* have been observed to cause a number of diseases including autosomal dominant EDMD and X-linked EDMD (X-EDMD), respectively [26]. EDMD is characterized by skeletal muscle wasting, cardiac conduction defects and cardiomyopathy [26]. Interestingly, 60% of cases of EDMD do not have mutations in *LMNA* or *EMD*. Recently, mutations in both Nesprin 1 and 2 were observed to cause EDMD phenotypes [27]. Furthermore, Nesprin 1α mutations were observed in patients with non-ischemic dilated cardiomyopathy (DCM) [28]. Isolated skin fibroblasts from these patients were also observed to have altered nuclear shape [27], [28]. These data indicate that Nesprins may also play a role in cardiac pathology.

Taken together, these data lead to the intriguing possibility that Nesprins could play a role in nuclear function, mechanotransduction, cardiac function and pathology. However, the roles of Nesprins, in particular Nesprin 1 and 2, in cardiomyocyte mechanotransduction and overall cardiac function are largely unknown. Studies as to the function of Nesprin 1 and 2 in cardiomyocytes have been limited, given that dual global ablation of both factors results in postnatal lethality with respiratory defects [29]. Thus, to elucidate the role of Nesprin 1 and 2 in cardiomyocytes, we generated a number of knockout mouse lines in which Nesprin 1 and/or Nesprin 2 are ablated in cardiomyocytes. We demonstrate that ablation of both Nesprin 1 and 2 causes an early onset cardiomyopathy. Moreover we show that Nesprin 1 and 2 play a key role in the integration of biomechanical loading in cardiomyocytes. Taken together, analysis of these targeted ablation models have revealed that Nesprin 1 and 2 play critical roles in cardiomyocyte mechanobiology.

## Results

### Establishment of Nesprin 1 and 2 global null or cardiomyocyte specific knockout mice

To examine the role(s) of Nesprin 1 and 2, we generated a number of mouse lines in which all KASH-domain containing isoforms were floxed. Nesprin 1 global knockout mice were generated as previously described [13]. These mice were observed to not have a cardiac phenotype at one year of age, but did have severe skeletal muscle defects and some postnatal lethality [13]. Nesprin 2 global knockout mice were developed as previously described [13]. Briefly, we floxed the 7^th^ exon counting backwards from the last exon of Nesprin 2 (Supplemental Figure. S1A). Targeted ES cells were confirmed using Southern blot analyses (Supplemental Figure. S1B). Chimeric males were bred to Black Swiss females to generate *nesprin2^f/+;neo+^* mice. These mice were then crossed to *Protamine-cre* mice and bred to homozygosity as previously described to generate global knockout mice (*nesprin 2^−/−^*) [30]. Deletion of Nesprin 2 was confirmed via PCR from tails, semi-quantitative PCR from hearts and Real Time PCR from isolated cardiomyocytes (Supplemental Figure. S1C,D,G). Deletion was confirmed at the protein level via immunostaining in both cardiac fibroblasts and myocytes (Supplemental Figure. S1E,F). These mice were viable and did not present cardiac defects (see below), skeletal muscle defects (data not shown) or lethality at 1 year of age.

It has been reported the global ablation of both Nesprin 1 and Nesprin 2 results in neonatal lethality with respiratory defects [29]. To investigate the specific role of Nesprin 1 and/or 2 in myocytes, we established mouse lines in which one or both factors were ablated in cardiomyocytes. To minimize incomplete Cre-mediated excision in the presence of four floxed alleles, we generated Nesprin 1 and 2 double knockout mice with cardiac specific Nesprin 1 deletion, using mice which were positive for *Nkx2.5Cre* [31], [32], homozygous floxed for Nesprin 1, and germline homozygous null for Nesprin 2 (*nesprin 1^f/f^;nesprin 2^−/−^;Nkx2.5Cre*). Excision of Nesprin 1 was observed to be ∼88±5% (n = 4 hearts) using isolated adult cardiomyocytes (Supplemental Figure S2). *nesprin 1^f/f^;nesprin 2^−/−^;Nkx2.5Cre* knockout mice were observed to be viable and born at normal Mendelian ratios. These mice also did not have any significant lethality at 1 year of age, compared to Wild Type controls. *nesprin 1^f/f^;nesprin 2^+/+^;Nkx2.5Cre* and *nesprin 1^f/f^;nesprin 2^−/−^* mice were used to model single deletion of Nesprin 1 or 2 respectively.

### Dual Ablation of Nesprin 1 and 2 in myocardium results in early onset cardiomyopathy

**Figure 1:**
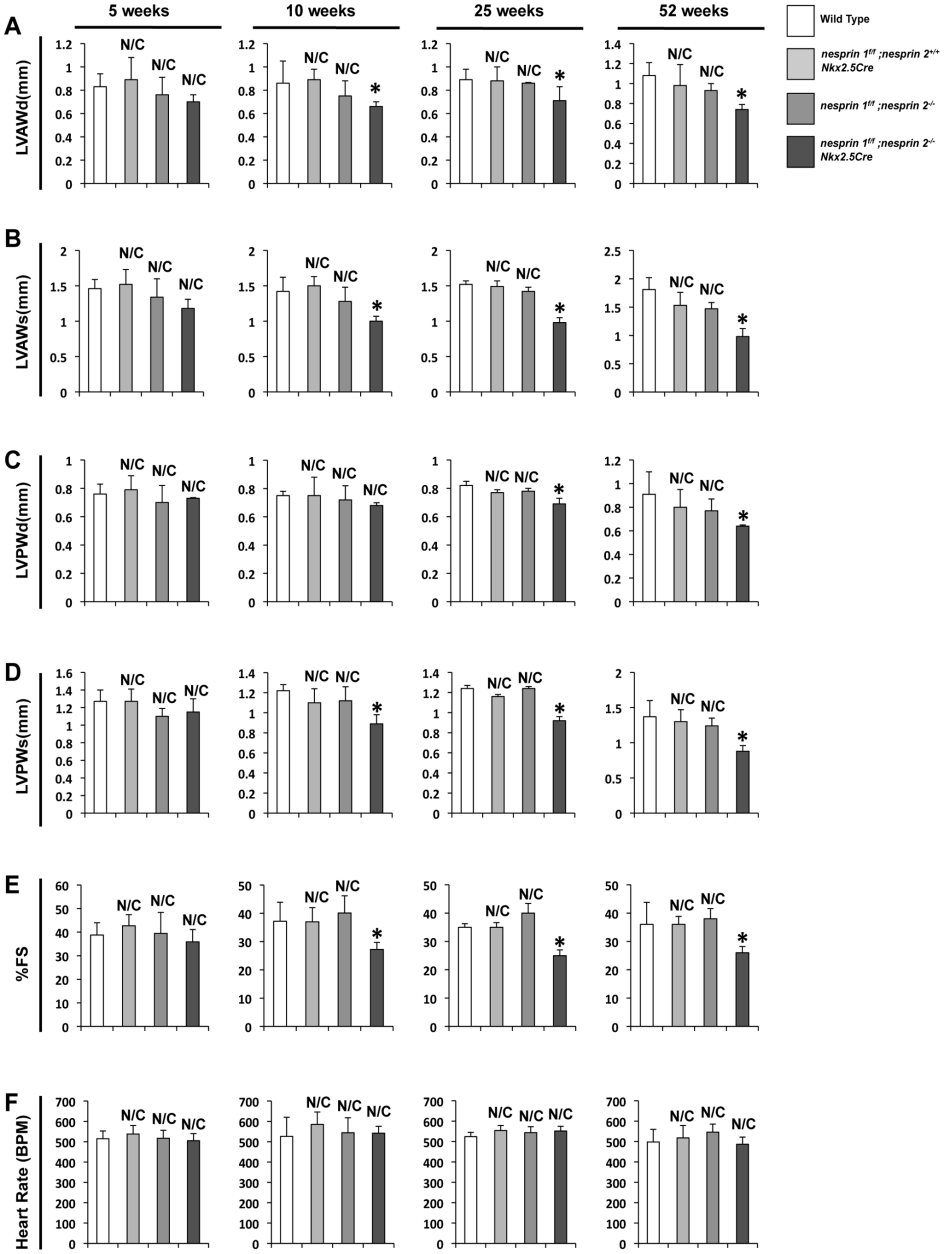
Deletion of both Nesprin 1 and 2 causes decreased cardiac wall thickness and impaired cardiac function. (A–D) Left ventricular anterior wall during diastole and systole (LVAWd and LVAWs), Left ventricular posterior wall during diastole and systole (LVPWd and LVPWs) at 5, 10, 25 and 52 weeks. (E) % Fractional Shortening (%FS) at 5, 10, 25, 52 weeks. (F) Heart Rate (HR) at 5, 10, 25, 52 weeks. N = 6–10 mice per condition/timepoint. ANOVA with a post hoc Bonferroni test *p<0.05, N/C = No significant change vs. Wild Type.

As demonstrated by our previous studies, global Nesprin 1 knockout mice do not have a cardiac phenotype [13]. These data suggest that there is a potential overlap of function of both Nesprin 1 and 2. We first examined effects of ablation of both Nesprins 1 and 2 on cardiac function, via echocardiographic analyses. Mice from all four conditions were examined at 5, 10, 25 and 52 weeks of age. Wild Type, *nesprin 1^f/f^;nesprin 2^+/+^;Nkx2.5Cre* and *nesprin 1^f/f^;nesprin 2^−/−^* mice did not have any functional defects over the time course examined (Figure 1). At 5 weeks of age, *nesprin 1^f/f^;nesprin 2^−/−^;Nkx2.5Cre* were not observed to have any cardiac functional defects or myocardial wall thinning (Figure 1). Examination at 10 weeks of age found that *nesprin 1^f/f^;nesprin 2^−/−^;Nkx2.5Cre* mice had decreased wall thickness; measured by left ventricular anterior wall during diastole and systole (LVAWd and LVAWs), left ventricular posterior wall during diastole and systole (LVPWd and LVPWs ) (Figure 1A–D) and diminished fractional shortening (Figure 1E). Examination at 25 weeks and 52 weeks found further decrease in fractional shortening with some decrease in wall thickness. Heart rates were not changed in these mice over the observed time course (Figure 1F).

**Figure 2:**
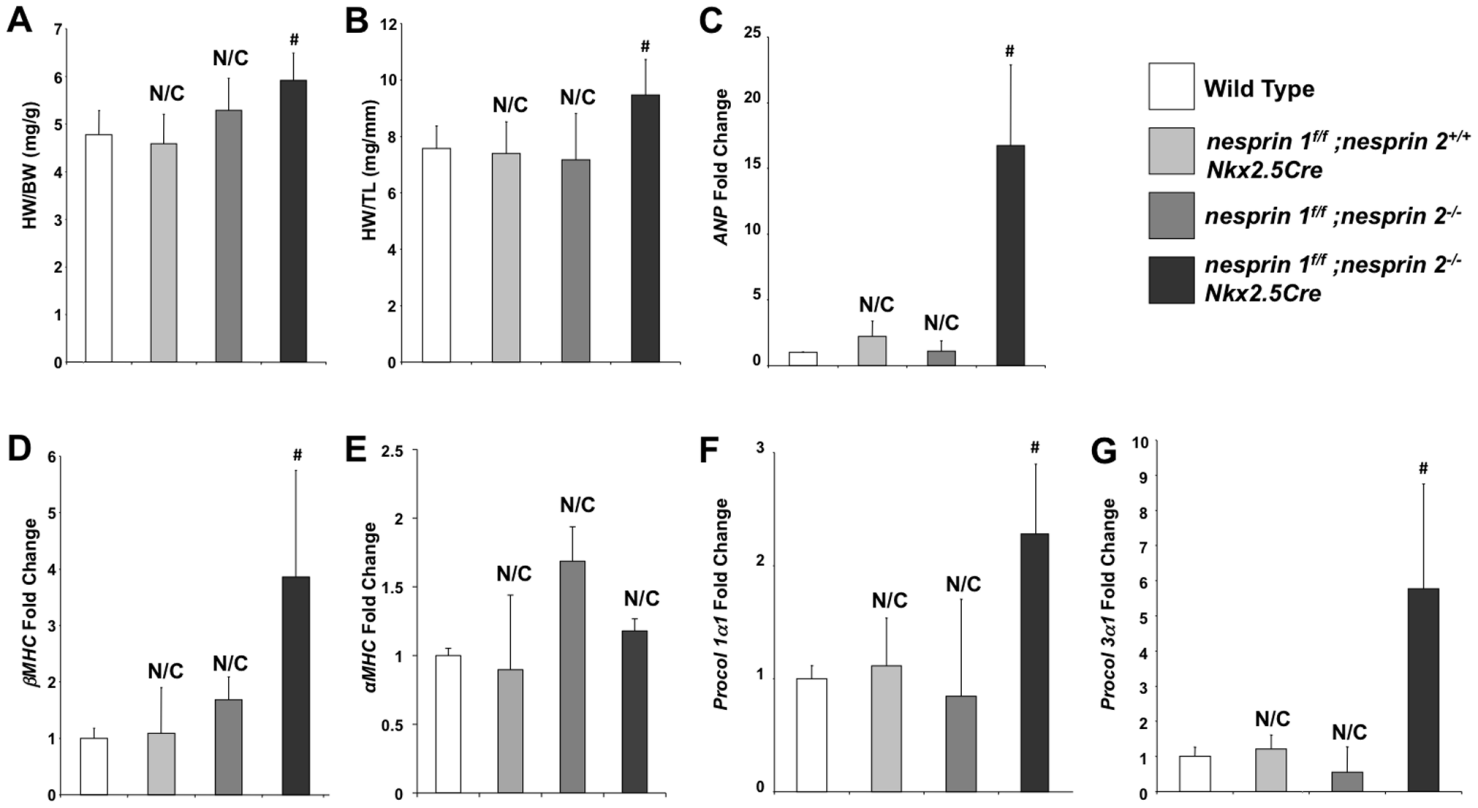
Ablation both Nesprin 1 and 2 alters cardiac morphology, reactivates fetal gene program and activates profibrotic genes. (A) and (B), 10 week old mice hearts: Morphological analyses of heart weight (HW)/body weight (BW) or HW/tibia length(TL). N = 10–11samples per condition (C–F), Real Time PCR analyses of mRNA expression levels for (C) *ANP* (D) *ßMHC* (E) *αMHC* (F) *Procol1α1* (G) *Procol3α1* N = 3–4 per condition. N/C = No significant change, ANOVA with a post hoc Bonferroni test #p<0.01.

**Figure 3:**
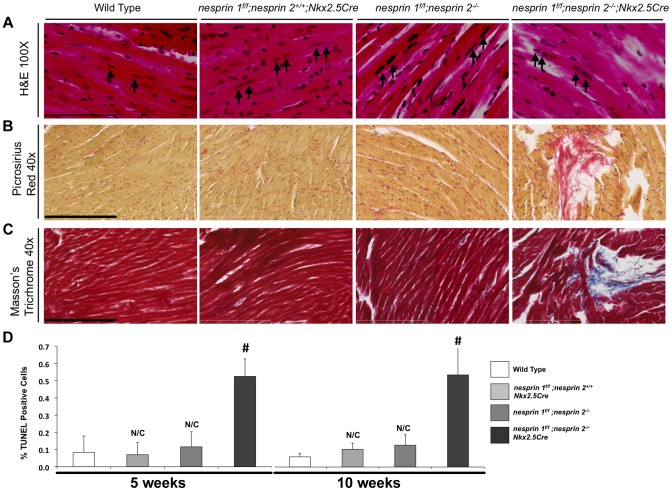
Histological and TUNEL analyses of Nesprin 1 and/or 2 ablation. (A) Representative 100× H&E images of indicated hearts. Black arrows = Myocyte nuclei. Black bar = 80 µm. (B) and (C), Representative 40× images of (B), Picrosirius Red (Brown = nuclei, red = collagen) or (C) Masson Trichrome (Red = Myocardium, Blue = Collagen, Black = Nuclei). Black bar = 200 µm. (D) TUNEL analyses of Nesprin 1 and/or 2 knockout hearts at 5 Weeks or 10 Weeks. N = 3 per condition. N/C = No significant change, ANOVA with a post hoc Bonferroni test #p<0.01.

Given the early onset of cardiac dysfunction in *nesprin 1^f/f^;nesprin2^−/−^;Nkx2.5Cre* mice at 10 weeks of age, we performed detailed analyses of these hearts at 10 weeks. Examination of HW/BW and HW/TL ratios at this stage revealed significant increases in *nesprin 1^f/f^;nesprin2^−/−^;Nkx2.5Cre* hearts compared to Wild Type, *nesprin 1^f/f^;nesprin 2^+/+^;Nkx2.5Cre* and *nesprin 1^f/f^;nesprin 2^−/−^* (Figure 2A,B). Real Time PCR analyses of fetal gene expression also found significant increases in *ANP* and *ßMHC* in *nesprin 1^f/f^;nesprin2^−/−^;Nkx2.5Cre* hearts (Figure 2C,D). No change in *αMHC* mRNA expression was observed (Figure 2E). Moreover, examination of pro-fibrotic genes *procollagen1α1* and *procollagen3α1* also observed significant increases in hearts from the latter group (Figure 2F,G). To further characterize cardiac pathology, we next performed Masson Trichrome and Picrosirius Red examination of *nesprin 1^f/f^;nesprin2^−/−^;Nkx2.5Cre* hearts. Histological analyses of hearts at 10 weeks of age also revealed fibrosis in *nesprin 1^f/f^;nesprin2^−/−^;Nkx2.5Cre* hearts when compared to Wild Type, *nesprin 1^f/f^;nesprin 2^+/+^;Nkx2.5Cre* and *nesprin 1^f/f^;nesprin 2^−/−^* hearts (Figure 3B,C). We also probed our model to examine cell death and observed increased apoptosis as measured by TUNEL analyses at both 5 and 10 weeks in our *nesprin 1^f/f^;nesprin2^−/−^;Nkx2.5Cre* hearts (Figure 3D, Supplemental Figure. S3).

### Deletion of both Nesprin 1 and 2 causes altered cardiac nuclear position and shape

Previous examination of *nesprin 1* global knockout mice found altered skeletal muscle nuclear positioning [13]. In additional studies, mice with a 61 amino-acid substitution of the KASH domain of Nesprin 1 were observed to have elongated nuclei as examined by H&E sectioning [28]. Moreover, examination of nuclei in fibroblasts from patients with mutations in Nesprin 1 and/or 2 found significant morphological defects in nuclear shape [27]–[29]. Therefore, we performed detailed position and shape analyses of cardiomyocyte nuclei following ablation of Nesprin 1 and/or 2. Nesprin 1 or 2 global knockout mice were crossed into the Obscurin-H2B-GFP line, allowing for examination of all cardiomyocyte nuclei [33]. Qualitative vibratome thick section (∼30 µm) analyses revealed that loss of Nesprin 1 or 2 caused an elongation of cardiomyocyte nuclei (Supplemental Figure S4). Examination of H&E sections from Wild Type, *nesprin 1^f/f^;nesprin 2^+/+^;Nkx2.5Cre* and *nesprin 1^f/f^;nesprin 2^−/−^* hearts also showed elongation and positional changes in myocyte nuclei, as indicated by black arrows (Figure. 3A). These changes were exacerbated in *nesprin 1^f/f^;nesprin 2^−/−^;Nkx2.5Cre* hearts also as indicated by black arrows (Figure 3A).

**Figure 4:**
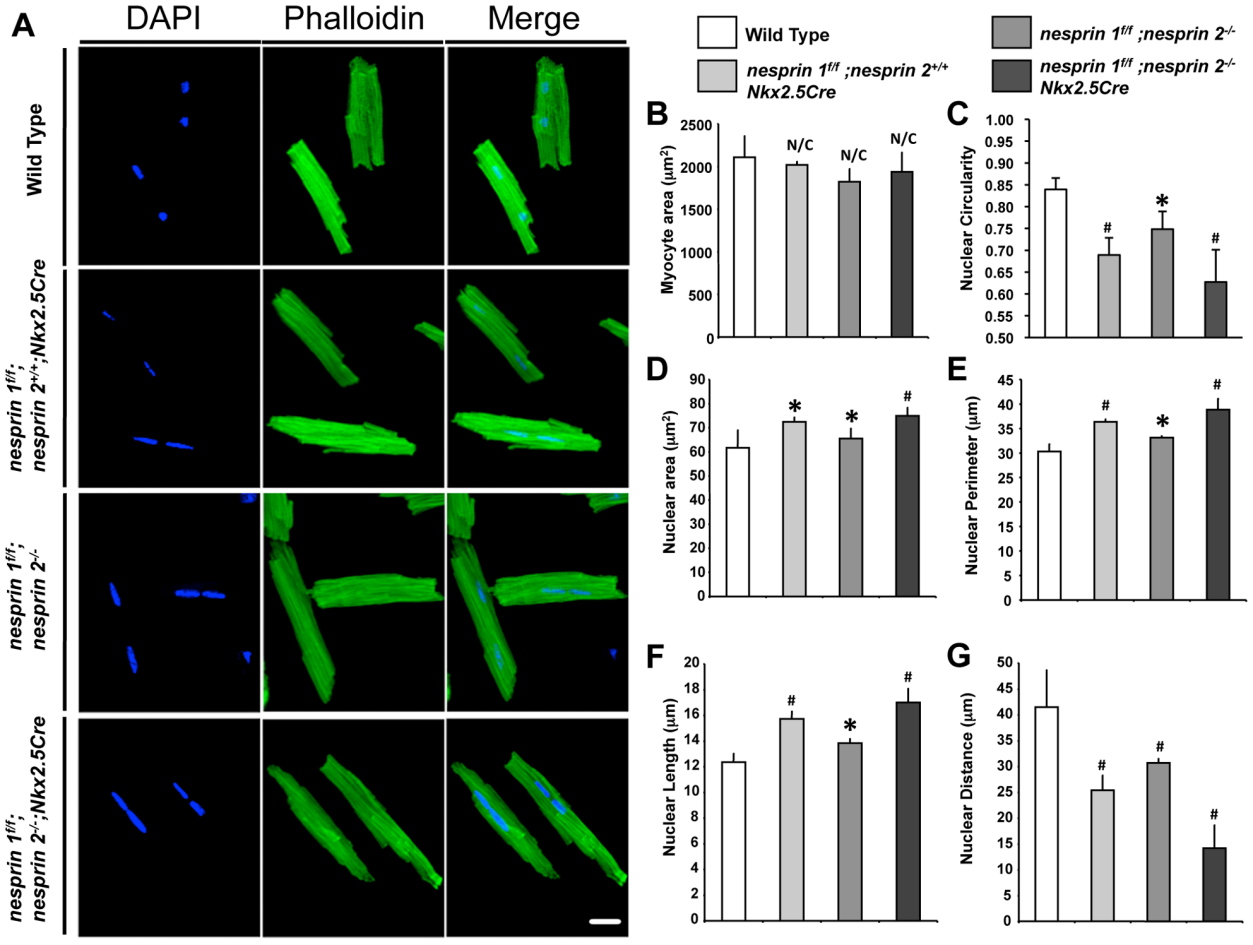
Cardiomyocyte nuclear morphology is altered in response to loss of Nesprin 1 and/or 2. (A) Representative 40× images of isolated cardiomyocytes (Blue = DAPI, Green = Phalloidin). White bar = 15 µm. (B–G) Morphological analyses of cardiomyocytes examining (B) Myocyte area (C) Nuclear circularity (D) Nuclear area (E) Nuclear perimeter (F) Nuclear length or (G) Nuclear distance. N = 200–300 cells per condition, from 3–4 mice. ANOVA with a post hoc Bonferroni test *p<0.05, #p<0.01.

To quantitate morphological differences observed in cardiomyocyte nuclei following ablation of Nesprin 1 and/or 2 we isolated cardiomyocytes from 10-week-old hearts and examined morphology using immunofluorescent and computational morphometric analyses (Figure 4A,D–F). Loss of either Nesprin 1 or 2 (*nesprin 1^f/f^;nesprin 2^+/+^;Nkx2.5Cre* and *nesprin 1^f/f^;nesprin 2^−/−^*) resulted in increased nuclear area, perimeter and length when compared to nuclei in Wild Type controls (Figure 4D–F). This defect was intensified in *nesprin 1^f/f^;nesprin2^−/−^;Nkx2.5Cre* cells (Figure 4D–F). Moreover, *nesprin 1^f/f^;nesprin 2^−/−^;Nkx2.5Cre* nuclei also appeared to be less circular than Wild Type controls. To quantify this we examined circularity of nuclei as previously described [34]. Here loss of either Nesprin 1 or 2 (*nesprin 1^f/f^;nesprin 2^+/+^;Nkx2.5Cre* and *nesprin 1^f/f^;nesprin 2^−/−^*) was found to decrease nuclear circularity. Loss of both Nesprin 1 and 2 (*nesprin 1^f/f^;nesprin2^−/−^;Nkx2.5Cre*) also intensified loss of circularity in these nuclei (Figure 4C). No change in myocyte area was observed (Figure 4B).

Studies of the murine heart have shown that ∼95 to 98% of cardiomyocytes are binucleated [35], [36]. Loss of Nesprin 1 and/or 2 appeared to decrease the distance between the two nuclei (Figure 3A, Supplemental Figure S3). To quantify this, centroid position was determined for each nuclei and distance from the center of one nucleus to the center of the other was determined. Distance between nuclei was significantly decreased with loss of either Nesprin 1 or 2 (*nesprin 1^f/f^;nesprin 2^+/+^;Nkx2.5Cre* and *nesprin 1^f/f^;nesprin 2^−/−^*). Inter-nuclear distance decreased from 41 µm±7.1 in Wild Type cells to 25 µm±2.8 or 30 µm±1.0 in *nesprin 1^f/f^;nesprin 2^+/+^;Nkx2.5Cre* and *nesprin 1^f/f^;nesprin 2^−/−^* cells respectively (Figure 4A,G). Again, loss of both Nesprin alleles (*nesprin 1^f/f^;nesprin 2^−/−^;Nkx2.5Cre*) resulted in a more significant decrease in inter-nuclear distance, to 12 µm±4.0 (Figure 4G).

### Ultrastructural nuclear abnormalities in Nesprin 1 and 2 dual ablation hearts

**Figure 5:**
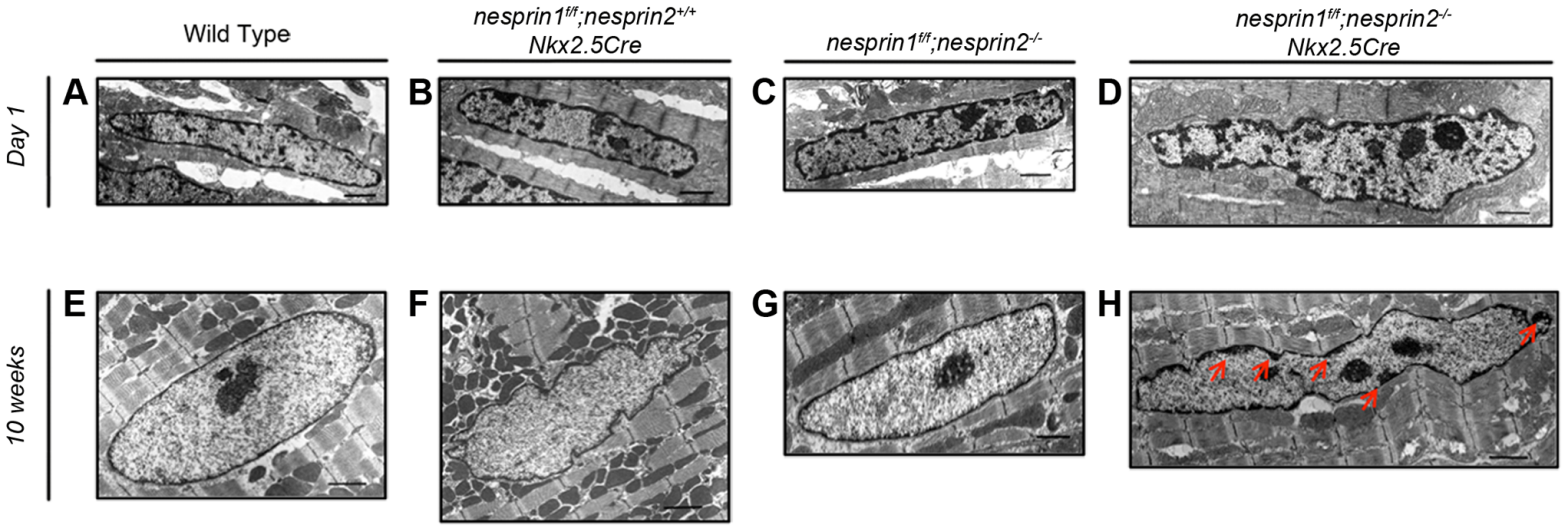
Examination of cardiomyocyte nuclear ultrastructure in response to loss of Nesprin 1 and/or 2. (A–D) Representative images of postnatal day 1 myocyte nuclei from (A) Wild Type (B) *nesprin 1^f/f^;nesprin 2^+/+^;Nkx2.5Cre* (C),*nesprin 1^f/f^;nesprin 2^−/−^* or (D), *nesprin 1^f/f^;nesprin 2^−/−^;Nkx2.5Cre* hearts. (E–H) Representative images of 10 week old myocyte nuclei from (E) Wild Type (F) *nesprin 1^f/f^;nesprin 2^+/+^;Nkx2.5Cre* (G) *nesprin 1^f/f^;nesprin 2^−/−^* or (H) *nesprin 1^f/f^;nesprin 2^−/−^;Nkx2.5Cre* hearts. Black Bars = 1 µm. Red Arrows highlight electron dense heterochromatin defects in 10 week old *nesprin 1^f/f^;nesprin 2^−/−^;Nkx2.5Cre hearts*.

Given observed defects in nuclear position and circularity, we further investigated the morphology of *nesprin 1^f/f^;nesprin2^−/−^;Nkx2.5Cre* nuclei. We first examined nuclei at postnatal day 1, prior to binucleation of cardiomyocytes [35]. Postnatal day 1 (P1) cardiomyocyte nuclei did not appear to be altered in *nesprin 1^f/f^;nesprin 2^+/+^;Nkx2.5Cre* and *nesprin 1^f/f^;nesprin 2^−/−^* groups compared to Wild Type (Figure 5A–C). Nuclei appeared to be oval shaped with electron dense regions of similar size at the periphery. This suggests that chromatin localization was not altered at P1 in response to loss of Nesprin 1 or 2. Examination of P1 *nesprin 1^f/f^;nesprin 2^−/−^;Nkx2.5Cre* nuclei found these nuclei did appear to have altered morphology with a more irregular shape compared to Wild Type controls (Figure 5D). Interestingly, *nesprin 1^f/f^;nesprin 2^−/−^;Nkx2.5Cre* cardiomyocyte nuclei did not appear to have an increased number of electron dense regions, suggesting that chromatin localization was not altered at P1 in dual Nesprin 1 and 2 knockout hearts.

We then examined cardiomyocyte nuclei by electron microscopy at 10 weeks, after the onset of cardiac dysfunction and binucleation [35]. At 10 weeks of age, Wild Type cardiomyocyte nuclei appeared to be oval in shape with a thin peripheral electron dense heterochromatin proximal to the INM (Figure 5E). Nesprin 1 ablated (*nesprin 1^f/f^;nesprin 2^+/+^;Nkx2.5Cre*) nuclei had an increased number of invaginations and increased perimeter, in agreement with immunofluorescent analyses. Again, these nuclei did not have altered electron dense heterochromatin at the INM (Figure 5F). Nesprin 2 knockout (*nesprin 1^f/f^;nesprin 2^−/−^*) nuclei also appeared elongated with increased perimeter, but not an increase number of invaginations (Figure 5G). These nuclei also did not have any alterations in electron dense heterochromatin at the INM (Figure 5G). Interestingly, ablation of both Nesprin 1 or 2 (*nesprin 1^f/f^;nesprin 2^−/−^;Nkx2.5Cre*) resulted in significantly altered nuclear appearance. These nuclei were elongated compared to Wild Type controls, with regions of electron dense chromatin distal to the INM (red arrows, Figure 5H).

### Dual ablation of Nesprin 1 and 2 did not alter expression, but did alter localization, of LINC complex or LINC complex associated factors

**Figure 6:**
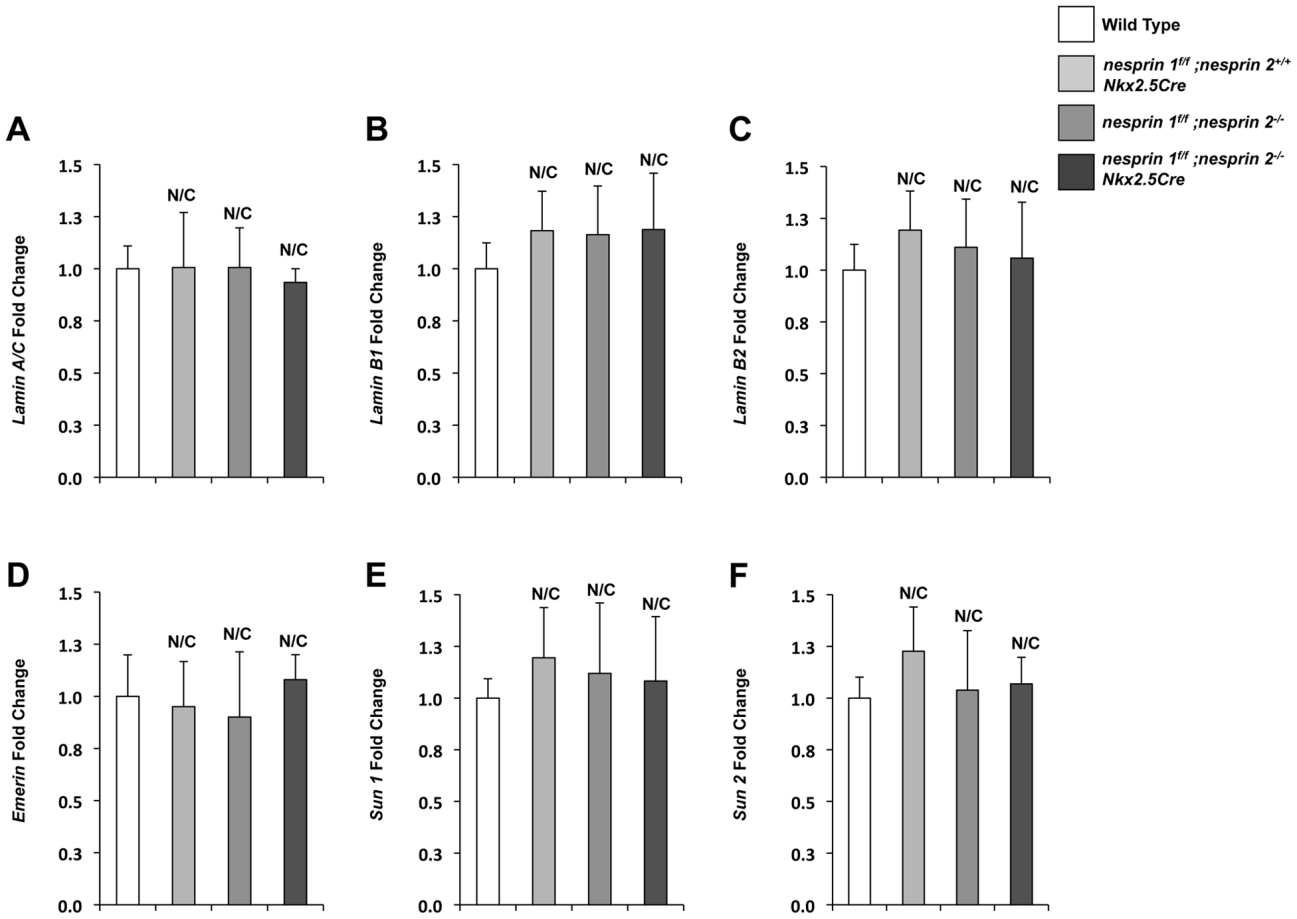
Real Time PCR analyses of LINC complex and LINC complex associated factors. (A–E) Real Time PCR analyses of (A) *Lamin A/C* (B) *Lamin B1* (C)*Lamin B2* (D)*Emerin* (E)*SUN1* (F)*SUN2*. Real Time PCR analyses are representative of N = 4–5 cardiomyocyte isolations. ANOVA with a post hoc Bonferroni test. N/C = No significant change.

KASH domain containing isoforms of both Nesprin 1 and 2 have been shown to coimmunoprecipitate and colocalize with Lamin A/C and Emerin in fibroblasts and skeletal muscle [9], [15], [37], [38]. Conversely, loss of Lamin A/C in deletion and mutant fibroblasts and COS7 cells have been shown to disrupt localization of both Nesprin 1 and 2 from the nuclear envelope [15], [39], [40]. Moreover, EDMD fibroblasts with mutations in Nesprin 1α1, Nesprin 1 or Nesprin 2 were also observed to have disrupted Lamin A/C and Emerin localization [17], [41]. Given these data, we examined the LINC complex associated factors (Lamin A/C, B1,B2, Emerin) as well as the LINC complex factors (SUN 1 and 2) to look for changes in expression levels. Both Western blot and Real Time PCR analyses revealed that expression levels of LINC Complex and LINC complex associated factors were not changed at the protein (Supplemental Figure S5) or mRNA levels (Figure 6A–F). Analyses of localization of SUN 2 did not reveal any changes in localization (Supplemental Figure S6). However, loss of both Nesprin 1 and 2 had a significant impact on localization patterns of both Lamin A/C and Emerin (Supplemental Figures S7 and S8).

### Deletion of Nesprin 1 and 2 abrogated the nuclear biomechanical gene response

The nucleus has been observed to be a critical integration point of mechanotransduction, regulating gene expression and cellular function [42]. Previous studies have indicated that the mechanotransduction response is abrogated following mutation or deletion of LINC complex associated factors, Lamin A/C and Emerin [21], [22], [43]. However, the role(s) of Nesprin 1 and/or 2 in the integration of mechanotransduction within the cardiomyocyte is unknown. To examine this, we isolated day 1 to 2 neonatal cardiomyocytes from Wild Type, *nesprin 1^f/f^;nesprin 2^+/+^;Nkx2.5Cre*, *nesprin 1^f/f^;nesprin 2^−/−^* and *nesprin 1^f/f^;nesprin2^−/−^;Nkx2.5Cre* hearts. These cells were plated onto micropatterned membranes to ensure *in vivo*-like cell morphology and rod-like orientation (Supplemental Figure S9). We then interrogated neonatal cardiomyocytes with a strain regime that recapitulated *in vivo* loading conditions, as per the literature [44], [45]. Indeed, previous research from our group has shown that anisotropic loading of neonatal rat cardiomyocytes induces increases in expression of the known hypertrophic markers [46]. Therefore, anisotropic biaxial loading was applied to 14% Lagrangian strain along the culture transverse axis, and 3.6% along the culture longitudinal axis using custom stretchers as previously described [47], [48].

**Figure 7:**
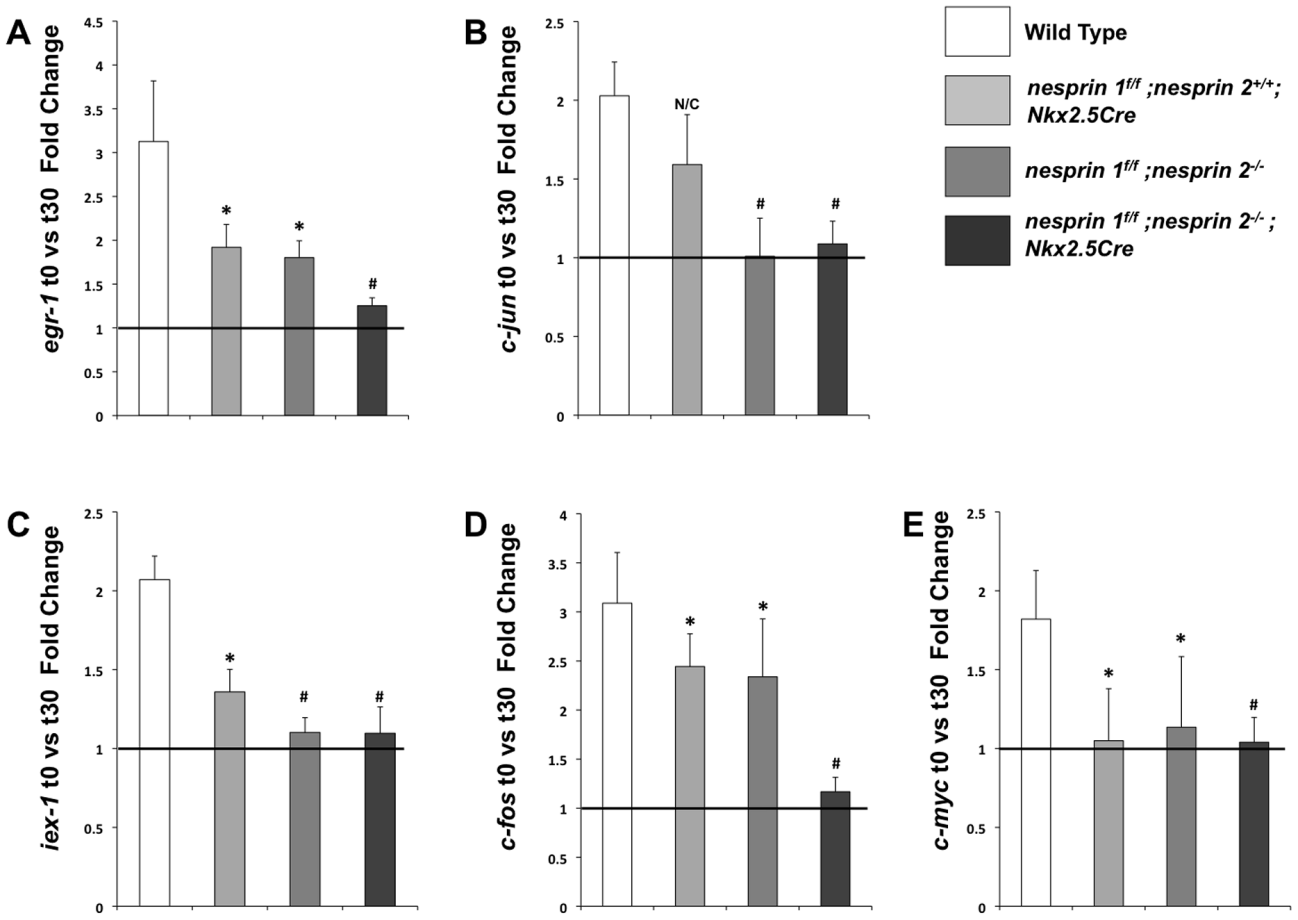
Examination of biomechanical gene response in isolated neonatal cardiomyocytes from Wild Type, *nesprin 1^f/f^;nesprin 2^+/+^; Nkx2.5Cre*, *nesprin 1^f/f^;nesprin 2^−/−^* or *nesprin 1^f/f^;nesprin 2^−/−^;Nkx2.5Cre hearts*. (A–E)Real Time PCR analyses of mRNA expression levels for (A) *egr-1* (B) *c-jun* (C) *iex-1* (D) *c-fos* and (E) *c-myc*. N = 3–5 stretchers/isolations per condition. ANOVA with a post hoc Bonferroni test N/C = No significant change, *p<0.05, #p<0.01.

Examination of known biomechanical response genes (*egr-1*, *iex-1*, *c-jun*, *c-fos and c-myc*) [21], [22], [49] via Real Time PCR found increased levels of these factors after 30 min of load in Wild Type cardiomyocytes (Figure 7A–E). Nesprin 1 loss (*nesprin 1^f/f^;nesprin 2^+/+^;Nkx2.5Cre*) resulted in a blunted response in levels of *egr-1*, *iex-1*, *c-jun and c-fos* mRNAs, with no alterations in response of *c-myc* mRNA. Nesprin 2 loss (*nesprin 1^f/f^;nesprin 2^−/−^*) resulted in blunted response of *egr-1*, *iex-1* and *c-fos* mRNAs with no alterations in response of *c-myc* or *c-jun* mRNAs. Interestingly, increased expression of all factors (*egr-1*, *iex-1*, *c-jun*, *c-fos and c-myc*) were significantly abrogated in *nesprin 1^f/f^;nesprin 2^−/−^;Nkx2.5Cre* cardiomyocytes (Figure 7A–E). These data suggested that while the biomechanical response is impaired with the loss of Nesprin 1 or 2, it is strongly abrogated in the absence of both Nesprins. This indicates that either Nesprin 1 or 2 partially compensates for the loss of each factor in single knockouts. Furthermore, these data suggest that Nesprins 1 and/or 2 play a significant role in the biomechanical function of the cardiomyocyte nucleus.

## Discussion

A network of molecular factors act to transmit mechanical cues from the ECM to the cytoskeleton [2]. These mechanical forces are transduced throughout the cell by various structural proteins and affect the physiological functions of the organ, as well as organelle function and positioning; including the nucleus. Indeed, recent studies of the nucleus have suggested that it functions as a primary integration point for response and regulation of mechanical forces from the cell, and that alterations in nuclear shape or position could alter the global functions of the cell and organ [21], [22], [49], [50]. Thus structural factors that mediate the integration of biomechanical signals into the nucleus could lend significant insight into both the physiology and pathophysiology of the cardiomyocyte. Recent studies have highlighted the LINC complex, and in particular the Nesprins, as being important for nuclear mechanical function in isolated fibroblasts, clinical cardiac pathology and skeletal muscle function [13], [26]–[28]. Nesprins have also been implicated in mechanosensing in endothelial cells under shear stress [51]. Clinical studies have demonstrated that mutations in either Nesprins 1 or 2 can result in a number of cardiac pathologies and EDMD-like phenotypes [27], [28], [41]. Moreover, studies of isolated fibroblasts from Nesprins 1 or 2 mutations observed altered nuclear morphology and localization of the LINC complex associated factors [27], [28], [41]. Taken together, these studies have raised the intriguing possibility that Nesprins 1 and/or 2 could play critical role(s) in cardiac function, nuclear positioning and mechanotransduction. These types of studies in the heart have, however, been limited in ablation models due to early neonatal lethality in dual global Nesprin 1 and 2 double knockout mice [29]. In this study we examine the role of Nesprin 1 and/or 2 ablation(s) in a cardiac-targeted ablation model at the tissue and cellular level. Moreover, we sought to dissect the precise roles of these factors within the cardiomyocyte under in vitro biomechanical load.

Studies of Nesprin loss or mutation with regards to cardiac function have been focused on examination of Nesprin 1 KASH domain mutants and our global knockout mice. Mice where the KASH domain of Nesprin 1 was replaced by 61 alternate amino acids exhibited late onset (52 weeks) cardiomyopathy and conduction defects [27], [28]. Interestingly, we did not observe cardiac abnormalities in Nesprin 1 global knockouts at 52 weeks of age [13]. Despite the contradictory nature of these findings, both studies suggest that loss of Nesprin 1 alone is insufficient to cause early onset cardiomyopathy. These data also raise the possibility that another factor could compensate for the loss of Nesprin 1. Given the overlap in function as well as structure of both small and giant isoforms of Nesprins 1 and 2 [9], [15], [37], [38], we hypothesized that Nesprin 2 may act in a compensatory role in cardiac tissue function. Indeed, our data found that targeted cardiomyocyte ablation of Nesprin 1 and 2 caused an early onset of cardiac dysfunction with increased fibrosis and fetal gene re-expression (Figure 1–3). These data suggest that correct anchoring of the nucleus to the cytoskeleton is a key aspect of normal physiology and homeostasis.

Postnatal cardiomyocyte growth is characterized by unique growth patterns in response to increased hemodynamic load during postnatal life [52], [53]. During this process, cardiomyocytes transition from mononucleated to binucleated cells, primarily through karyokinesis without cytokinesis [35]. Given the dynamic nature of this process and the early onset of cardiomyopathy in *nesprin 1^f/f^;nesprin 2^−/−^;Nkx2.5Cre* hearts, we hypothesized that loss of Nesprin 1 and/or 2 would cause morphological and positional defects in cardiomyocyte nuclei. Our study clearly showed that ablation of these factors alters nuclear positioning. Quantification of nuclear morphology showed that at 10 weeks of age (Figure 4A–G) cardiomyocyte nuclei were elongated, less circular and closer together with single ablation of Nesprin 1 or 2. This phenotype was exacerbated in dual ablation of Nesprin 1 and 2, wherein nuclear shape and position were significantly altered upon comparison to Wild Type controls. Moreover, TEM analyses indicated that loss of both Nesprin 1 and 2 resulted in an elongated, morphologically altered nucleus (Figure 5). The observed changes suggested that while loss of either Nesprin 1 or 2 causes some nuclear positioning and morphological defects, loss of both Nesprin 1 and 2 results in more severe aberrations in normal positioning and morphological features of cardiomyocyte nuclei. In addition, these data also show that loss of Nesprin 1 and/or 2 also alters localization of heterochromatin and thus may have an effect on the epigenetic status of myocyte nuclei. This in turn could alter the transcriptome of the cell in response to normal contractile activity or in response to stress. Further examination of this phenomenon is needed in future studies.

Observed changes in nuclear morphology suggested that these cells may have deficiencies in nuclear function. The “structural hypothesis” of disease suggests that mutations in genes involved in the position and tension of the nucleus can alter biomechanical properties of the cell [1]. Force transmission, via the cytoskeleton to the nucleus is mediated by the elements of the LINC complex. Components of the LINC complex associated factors (Lamin A/C, Emerin) have been shown to mediate cellular response to mechanotransduction, as measured by expression of candidate genes (*egr-1*, *iex-1*, *c-fos*, *c-jun* and *c-myc*) [21], [22], [49]. Given that dual ablation of Nesprin 1 and 2 resulted in cardiac defects, and severe abnormalities in nuclear positioning, we hypothesized that ablation of Nesprin 1 and 2 would cause abrogation of the mechanotransduction response in cardiomyocytes.

Biomechanical stimulation of cardiomyocytes results in upregulation of biomechanically responsive genes, including *egr-1*, *iex-1*, *c-fos*, *c-jun* and *c-myc*. In contrast to Wild Type controls, Nesprin 1 and 2 knockout cardiomyocytes did not exhibit upregulation of this biomechanically responsive gene program in response to stretch. Interestingly, single ablation of Nesprin 1 or 2 caused selective decreases in responsiveness of some of these factors to biomechanical load. Our data also suggested that while there are some defects in Nesprin 1 or 2 single ablation cardiomyocytes in both nuclei and gene responses to load, these defects may not be enough to cause significant alterations in cardiac function.

**Figure 8:**
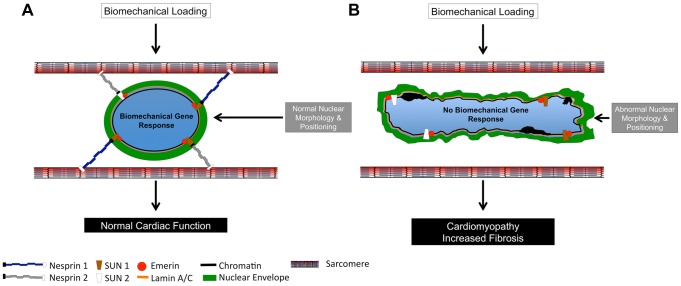
Model of Nesprin 1 and/or 2 loss in cardiomyocytes. (A and B) Diagrammatic representation of (A) Wild Type and (B) Nesprin 1 and 2 double knockout cardiomyocyte nuclei. (A) Wild Type nuclei are anchored to the sarcomeric structure via Nesprin 1 and 2. Application of 14% anisotropic loading results in normal cellular biomechanical gene response (*egr-1*, *c-jun*, *iex-1*, *c-fos* and *c-myc*.). Cardiac development and nuclear positioning/morphology are also normal in these cells. (B) Loss of both Nesprin 1 and 2 causes ablation of biomechanical gene response upon application of strain. Hearts from these mice develop cardiomyopathy with fibrosis, and have altered nuclear positioning and morphology.

Together, these data clearly demonstrated that Nesprins 1 and 2 play overlapping and essential roles in cardiac physiology, maintenance and responsiveness to biomechanical load. These data also suggested a model (Figure 8) where the presence of both Nesprin 1 and 2 is required for cardiomyocyte nuclear positioning and linkage to the cytoskeleton/sarcomere via the LINC complex and LINC complex associated factors. Failing to correctly establish these structures affects normal cardiomyocyte development and the capacity to respond to mechanical stress, leading to impaired cardiac function. Ablation of Nesprin 1 and 2 results in abnormal localization of other members of the LINC complex, Emerin and Lamin A/C. It is possible that localization or functions of other cytoskeletal proteins, which connect to the LINC complex, are also perturbed, which will be the subject of future studies. It should also be pointed out that Nesprin 1 and 2 are ubiquitously expressed and therefore it is likely that the role we have uncovered for Nesprin in transducing biomechanical signals into gene expression in the context of cardiomyocytes is likely to be more generally applicable to the role of Nesprin in multiple cell types.

## Materials and Methods

### Animal care

The UCSD Animal Care Personnel maintained all animals and the UCSD Institutional Animal Care and Use Committee approved all experimental procedures.

### Gene targeting and generation of Nesprin 2 knockout mice

Genomic DNA fragments of Nesprin 2 were amplified from R1 ES cells using polymerase chain reaction (PCR) and used to construct the Nesprin 2-targeting construct as previously described (Supplemental Figure. S1A) [31]. One LoxP site was inserted into intron 7, as counted backward from the last exon, and a second LoxP site, together with a neomycin cassette flanked by FRT sites, was inserted into intron 6, again as counted backward from the last exon. The targeting construct was verified by sequencing and linearized with the restriction enzyme NotI and electroporated into R1 ES cells derived from 129/SvJ mice (UCSD Transgenic and Gene Targeting Core, La Jolla, CA, USA). Targeted ES cells were identified by Sothern blotting analysis as previously described [54], [55]. Genomic DNA from G418-resistant ES cell clones was digested with the restriction enzyme EcoR V and hybridized with the radiolabeled 564 bp probe generated by PCR using mouse genomic DNAs and *nesprin 2* specific primers (forward, 5′-TCCAGACAACAGGCTACACTTTACC-3′ and reverse, 5′-CCAATTAAAGAATTGTAAATTTGACC-3′). The WT allele is represented as a 12.1 kb band, whereas a 10.5 kb band represents the targeted allele. ES cells from a homologous recombinant clone were then microinjected into C57BL/6 blastocysts. Male chimeras were bred with female Black Swiss mice (Taconic Inc., Hudson, NY, USA) to generate germline-transmitted heterozygous mice with a neo cassette, which were further confirmed by PCR and Southern blot analysis of mouse-tail DNA. The global heterozygous Nesprin 2 knockout mice were generated by crossing floxed heterozygous mice with the protamine-Cre transgenic mouse line as previously described [30].

### Semi-quantitative RT-PCR

Total RNA of various muscle tissues was isolated using Trizol (Invitrogen) according to the instructions of the manufacturer. RNA amounts were measured with a photospectrometer (Biorad) and equalized. Reverse transcriptase reaction was performed using oligo dT17 primer and the superscript II first-strand synthesis system (Invitrogen). Amplification of GAPDH for standardization was done using KOD DNA polymerase (EMD-Millipore). Bands were analyzed by agarose gel electrophoresis.

### Transmission electron microscopy

For TEM, mice were anaesthetized with Ketamine with Xylene, and subsequently perfused through the left ventricle with Tryode buffer including 50 mM KCl, followed by fixative (2% paraformaldehyde, 2% glutaraldehyde in PBS, pH 7.4). The heart were removed, diced, and kept in fixative overnight. The tissues were washed 3 times by 0.15 M sodium cacodylate buffer and then post-fixed with 2% OsO4 in 0.15 M sodium cacodylate buffer for one hour. The following day, tissue was stained overnight in 2% uranyl acetate, dehydrated, and embedded into Durcupan resin (EMD, Gibbstown, NJ) using a standard method. Ultra-thin sections (60–70 nm) were stained with 2% uranyl acetate and statured lead citrate solution, and observed with a JEOL-1200EX transmission electron microscope at an accelerating voltage of 80 kV.

### Nesprin 2 antibody

Rabbit antibody of Nesprin-2 was generated against the synthetic polypeptides: PREIQADSWRKRRES (6357–6371aa) (accession number NP_001005510) (Abgent, Ins., US). It is modified from human Nesprin 2 antibody [15]. Each polypeptide was conjugated to keyhole limpet haemocyanin and the conjugates injected into rabbits to produce polyclonal antibodies that were subsequently affinity purified and ELISA tested to confirm specificity.

### Micropatterning of substrate, *in vitro* analyses of biomechanical loading

Cardiomyocytes were cultured on a flexible, microgrooved substrate and subjected to sustained mechanical loading according to methods derived from previous studies [46]–[48]. Micropatterned topography was created using soft photolithography and polydimethylsiloxane (PDMS) molding to provide mechanical and extracellular matrix cues to cells on three sides and permit cell coupling across groove walls. Silicon wafer master molds were micropatterned with SU-8 2005 negative photoresist (MicroChem Corp., Newton, MA) using a custom photomask (Advance Reproductions Corp., North Andover, MA). Sylgard 186 PDMS, prepared at 10 parts base to 1 part curing agent, was spin-coated on the molds, degassed, and thermoset at 70 degrees for 30 minutes and at room temperature overnight. The microgrooved PDMS was mounted in custom cell stretchers and murine laminin (Sigma-Aldrich, St. Louis, MO) was adsorbed under 350 nm wavelength radiation at 10 µg/ml in phosphate buffered saline (PBS) (MediaTech, Manassas, VA). Excess protein was removed by rinsing twice in PBS prior to plating cells. The resulting 10 µm wide microgrooves were defined by walls 5 µm high and 10 µm wide. Anisotropic biaxial loading was applied to 14% Lagrangian strain along the culture transverse axis, and 3.6% along the culture longitudinal axis. The micropatterned cultures were incubated at 37°C and 10% CO_2_ during the 30 minute mechanical loading. Controls were subjected to placebo manipulations.

### Isolation of neonatal cardiomyocytes

Cardiomyocytes were isolated from 1–2 day old neonatal mouse hearts as previously described [56]. Cells were plated onto the microgrooved PDMS of the stretchers at a density of 1.5 million cardiomyocytes in an area of 1 square inch per stretcher. Myocytes were plated in a medium containing Dulbecco's modified Eagle medium and Medium 199 supplemented with 10% horse serum, 5% fetal bovine serum, 100 units/mL penicillin, and 100 µg/mL streptomycin, and incubated at 37°C and 10% CO_2_. At 72 hours after plating, media changed to a low-serum media containing 5% horse serum and 1% fetal bovine serum for another 24 hours prior to stretch.

### Real Time-PCR (RT-PCR) analysis

Samples from Wild Type, *nesprin 1^f/f^;nesprin 2^+/+^;Nkx2.5Cre*, *nesprin 1^f/f^;nesprin 2^−/−^* and *nesprin 1^f/f^;nesprin 2^−/−^;Nkx2.5Cre* hearts, isolated cardiomyocytes or stretch samples were homogenized in Trizol reagent (Invitrogen) or RNeasy Mini Kit (QIAGEN)to isolate total RNA. First strand cDNA was generated utilizing random hexamers and the Invitrogen Superscript II kit (Invitrogen). Primers for RT-PCR were used as previously described [31] or as listed in Supplementary Table 1. These primers were optimized using control murine cDNA (data not shown). RT-PCR reactions were performed using Sso-Fast EvaGreen Real Time PCR (BioRad) master mix in 96 well low profile PCR plates in the CFX96 BioRad thermocycler (BioRad).

### Paraffin processing, Histology analyses

Hearts were isolated from aged-matched Wild Type, *nesprin 1^f/f^;nesprin 2^+/+^;Nkx2.5Cre*, *nesprin 1^f/f^;nesprin 2^−/−^* and I hearts (10 week old mice). Adult hearts were perfused with phosphate buffered saline (PBS) supplemented with 50 mM potassium chloride and subsequently perfused and fixed in fresh 4% paraformaldehyde supplemented with 50 mM potassium chloride. Hearts were removed and placed overnight in 4% paraformaldehyde supplemented with 50 mM potassium chloride. Hearts were then processed using standard procedures and embedded into paraffin blocks for sectioning (10 µm). Paraffin sections were cut and stained according to Masson Trichrome, Picrosirius Red, or hematoxylin and eosin standard protocols as previously described [31]. Sections on the Hamamatsu NanoZoomer 2.0HT Slide Scanning System (Hamamatsu).

### Frozen processing, TUNEL staining and analyses

For frozen sections, hearts were fresh frozen using isopentane into frozen blocks (1∶1,OCT∶20%sucrose) and sections cut (7–10 µm). Frozen sections were then post fixed and samples stained first for α-Actinin (Abcam) and then for apoptosis using the In Situ Cell Death Detection kit (Roche) as per manufactures instructions. Images were taken using confocal microscopy (Olympus FV1000 Confocal) and images analyzed using ImagePro3D software analyses (MediaCybernetics).

### Langendorff perfusion of adult hearts

Dissociation of heart by Langendorff perfusion was performed as in prior studies [57]. Briefly, hearts we perfused with collagenase type II (Worthington) to dissociate the individual cardiomyocytes. Isolated cells were then filtered through a 100 µm mesh nylon filter before performing sequential sedimentation to enrich for myocytes. Isolated myocytes were then plated onto glass-covered dishes coated with Laminin (Sigma) for 1–2 hours.

### Echocardiographic analysis

Transthoracic echocardiography was performed on mice 5 to 52 weeks (adult) as previously described [31], [58]. Briefly, mice were anesthetized using 1% isoflurane. Mice were then moved to a biofeedback warming station. This was to maintained core body temperature. Mice were then placed under anesthesia (1 to .5% isoflurane). Ultrasound gel was applied to the chest of the animal. Echocardiography measurements then were obtained using the VEVO 2100 software package and ultrasound system with a linear transducer 45 MHz. (Visual Sonics, SonoSite). Images were processed and M-mode tracings were taken and measured for wall thicknesses and interdimensional space. This was done for both systole and diastole. Fractional shortening was calculated as previously described [58]. An average of six cardiac cycles per animal were analyzed. Six to ten animals were used per condition/per time point.

### Western blot analysis

Isolated cardiomyocytes from Wild Type, *nesprin 1^f/f^;nesprin 2^+/+^;Nkx2.5Cre*, *nesprin 1^f/f^;nesprin 2^−/−^* and *nesprin 1^f/f^;nesprin 2^−/−^;Nkx2.5Cre* hearts were lysed into buffer, run up and down into a syringe, quantified and placed directly into SDS-sample buffer. Samples were run on Bis-Tris gels (Invitrogen) and transferred to membranes. Membrane was stained using Ponceau, destained and then UV cross-linked for 10 min. Membranes were then blocked for 1 hour with 5% milk in TBST. Membranes were then probed with rabbit anti-Lamin A/C (a gift from Larry Gerace), anti-Lamin B1 (a gift from Larry Gerace), anti-Lamin B2 (a gift from Larry Gerace), anti-SUN 1 (Epitomics), anti-SUN2 (Sigma), anti-Emerin (Santa Cruz) or mouse anti-GAPDH (Santa Cruz) for 1 hour at room temperature. Blots were then washed three times in TBST and probed for 1 hour with secondary anti-rabbit or anti-mouse antibody. After washing of membranes three times in TBST for 10 minutes, membranes were developed for expression analyses.

### Immunofluorescent staining

For immunofluorescent staining, isolated adult cardiomyocytes were fixed for 30 min, room temperature with 2%PFA/PBS/0.1%Triton x-100. Samples were then stained with antibodies for Lamin A/C (A gift from Larry Gerace), Emerin (Santa Cruz), Sun2 (Sigma), α-actinin (Sigma) or Nesprin 2(Abgent). Samples were then stained with secondary antibodies conjugated to CY3 (Jackson ImmunoResearch Inc.). Samples were then incubated with DAPI (Invitrogen) and Phalloidin Alexa-488 (Invitrogen). Samples then imaged using standard confocal microscopy (Olympus FV1000 Confocal). Image analyses were then performed and samples quantified using either Velocity high performance 3D imaging software (PerkinElmer) or ImagePro3D software analyses (MediaCybernetics).

### Statistics

The data obtained from all analyses were measured for significance via Graphpad Prism by either student-t test or ANOVA with a post hoc Bonferroni test, P<0.05 was considered significant. All data are ± SEM.

## Supporting Information

Figure S1 Generation of Nesprin 2 all isoforms containing C-terminal domain knockout mice. (A) Targeting strategy. A restriction map of the relevant genomic region of Nesprin 2 is shown at the top, the targeting construct is shown in the middle, and the mutated locus after recombination is shown at the bottom. The grey box indicates an exon which is the 16th exon, as counted backward from the last exon, triangular black boxes indicate LoxP sites and rectangular grey boxes indicate frt sites. DTA, Diphtheria Toxin A chain gene, Neo, Neomycin resistance gene. (B) Detection of wildtype (WT) and knockout (KO) alleles by Southern blot analysis. DNA from electroporated ES cells was digested with EcoR V and analyzed by Southern Blot analysis with a probe as shown in A. The 12.1 kb and 10.5 kb bands represent WT and KO alleles, respectively. (C) PCR analysis of DNA isolated from tails of Nesprin 2 WT, heterozygous and homozygous KO mice. KO and WT mice show only one band using specific primers for the KO or WT allele, heterozygous mice show two bands. (D) RT-PCR analysis with RNA from muscle. No coding sequence of the deleted exon could be amplified from the KO sample. (E and F) Immunostaining from (E) Cardiac Fibroblasts and (F) Cardiomyocytes (Blue = DAPI, Red = Nesprin 2) Green bar = 10 µm, White Bar = 5 µm. Green arrows indicate nuclear membranes (G) Real Time PCR analyses of Nesprin 2 knockout from isolated cardiomyocytes. student-t test #P<0.01.
(TIF)

Figure S2 Validation of knockout of Nesprin 1 in cardiac specific deficient mice. Nesprin 1 Real Time PCR analyses of isolated cardiomyocytes from Wild Type, *nesprin1^f/f^;Nkx2.5Cre* and global *nesprin1^−/−^* mice. We observed an ∼88% decrease observed *nesprin1^f/f^;Nkx2.5Cre* isolated cardiomyocytes. student-t test #P<0.01.
(TIF)

Figure S3 Representative TUNEL stain in Nesprin 1 and/or 2 knockout hearts. Blue = DAPI, Green = TUNEL, Red = α-actinin White arrows indicate apoptotic nuclei.
(TIF)

Figure S4 Analyses of Nesprin 1 and Nesprin 2 knockout hearts. Representative 3D reconstruction, 30 µm thick images from (A) Wild Type;*Obs-H2bGFP* (B) *nesprin1^−/−^;Obs-h2b-GFP* and (C) *nesprin2^−/−^;Obs-h2b-GFP* Hearts. White arrows indicate cardiomyocyte nuclei. #p<0.01.
(TIF)

Figure S5 Western LINC complex and LINC complex associated factors. (A) Representative western blots for Lamin A/C, Lamin B1, Lamin B2, Emerin, SUN1, SUN2 and GAPDH. Western samples are representative of N = 3 cardiomyocyte isolations, (B–G) Quantification of Western analyses for LINC Complex and LINC Complex associated factors. ANOVA with a post hoc Bonferroni test N/C = No Change.
(TIF)

Figure S6 Loss of Nesprin 1 and 2 does not cause abnormal SUN 2 staining. (A) Representative immunofluorescence nuclear images from isolated adult cardiomyocytes (B) Quantification of percent cells with abnormal SUN 2 staining. Blue = DAPI, Red = SUN 2 ANOVA with a post hoc Bonferroni test #p<0.01. White Bar = 7 µm.
(TIF)

Figure S7 Loss of both Nesprin 1 and 2 had a significant impact on the localization patterns of Lamin A/C. (A) Representative immunofluorescence nuclear images from isolated adult cardiomyocytes (B) Quantification of percent cells with abnormal Lamin A/C staining. Blue = DAPI, Red = Lamin A/C ANOVA with a post hoc Bonferroni test #p<0.01. White Bar = 7 µm Yellow arrows = Abnormal Lamin A/C Stain.
(TIF)

Figure S8 Loss of both Nesprin 1 and 2 had a significant impact on the localization patterns of Emerin. (A) Representative immunofluorescence nuclear images from isolated adult cardiomyocytes (B) Quantification of percent cells with abnormal Emerin staining. Blue = DAPI, Red = Emerin ANOVA with a post hoc Bonferroni test #p<0.01. White Bar = 7 µm, Yellow arrows = Abnormal Emerin Stain.
(TIF)

Figure S9 Representative image of isolated cardiomyocytes on aligned, micropatterned substrates. Representative image of day 1–2 neonatal cardiomyocytes on aligned substrates. Cardiomyocytes appear rod-like and form cell-cell connections. (Green = α-actinin, Blue = DAPI, Red = Connexin-43).
(TIF)

Table S1 Real Time PCR Primers for study.
(DOCX)
