# Supplementary figures and images for: Targeted Ablation of Nesprin 1 and Nesprin 2 from Murine Myocardium Results in Cardiomyopathy, Altered Nuclear Morphology and Inhibition of the Biomechanical Gene Response
Source: PLoS Genet. 2014 Feb 20;10(2):e1004114. doi: 10.1371/journal.pgen.1004114 (PMC3930490; doi:10.1371/journal.pgen.1004114)

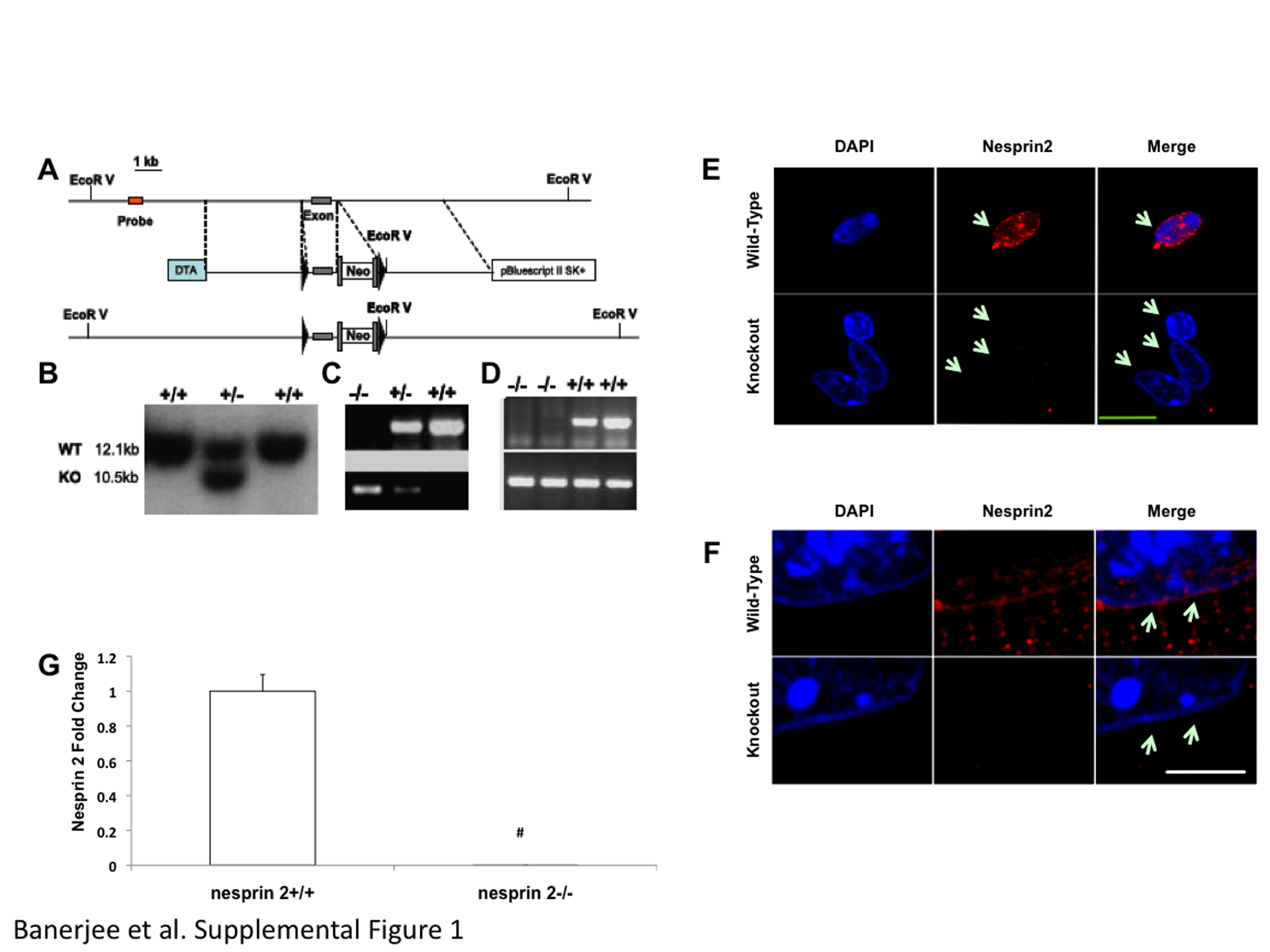

Supplement: Figure S1 — Generation of Nesprin 2 all isoforms containing C-terminal domain knockout mice. (A) Targeting strategy. A restriction map of the relevant genomic region of Nesprin 2 is shown at the top, the targeting construct is shown in the middle, and the mutated locus after recombination is shown at the bottom. The grey box indicates an exon which is the 16th exon, as counted backward from the last exon, triangular black boxes indicate LoxP sites and rectangular grey boxes indicate frt sites. DTA, Diphtheria Toxin A chain gene, Neo, Neomycin resistance gene. (B) Detection of wildtype (WT) and knockout (KO) alleles by Southern blot analysis. DNA from electroporated ES cells was digested with EcoR V and analyzed by Southern Blot analysis with a probe as shown in A. The 12.1 kb and 10.5 kb bands represent WT and KO alleles, respectively. (C) PCR analysis of DNA isolated from tails of Nesprin 2 WT, heterozygous and homozygous KO mice. KO and WT mice show only one band using specific primers for the KO or WT allele, heterozygous mice show two bands. (D) RT-PCR analysis with RNA from muscle. No coding sequence of the deleted exon could be amplified from the KO sample. (E and F) Immunostaining from (E) Cardiac Fibroblasts and (F) Cardiomyocytes (Blue = DAPI, Red = Nesprin 2) Green bar = 10 µm, White Bar = 5 µm. Green arrows indicate nuclear membranes (G) Real Time PCR analyses of Nesprin 2 knockout from isolated cardiomyocytes. student-t test #P<0.01. (TIF) [file pgen.1004114.s001.tif]

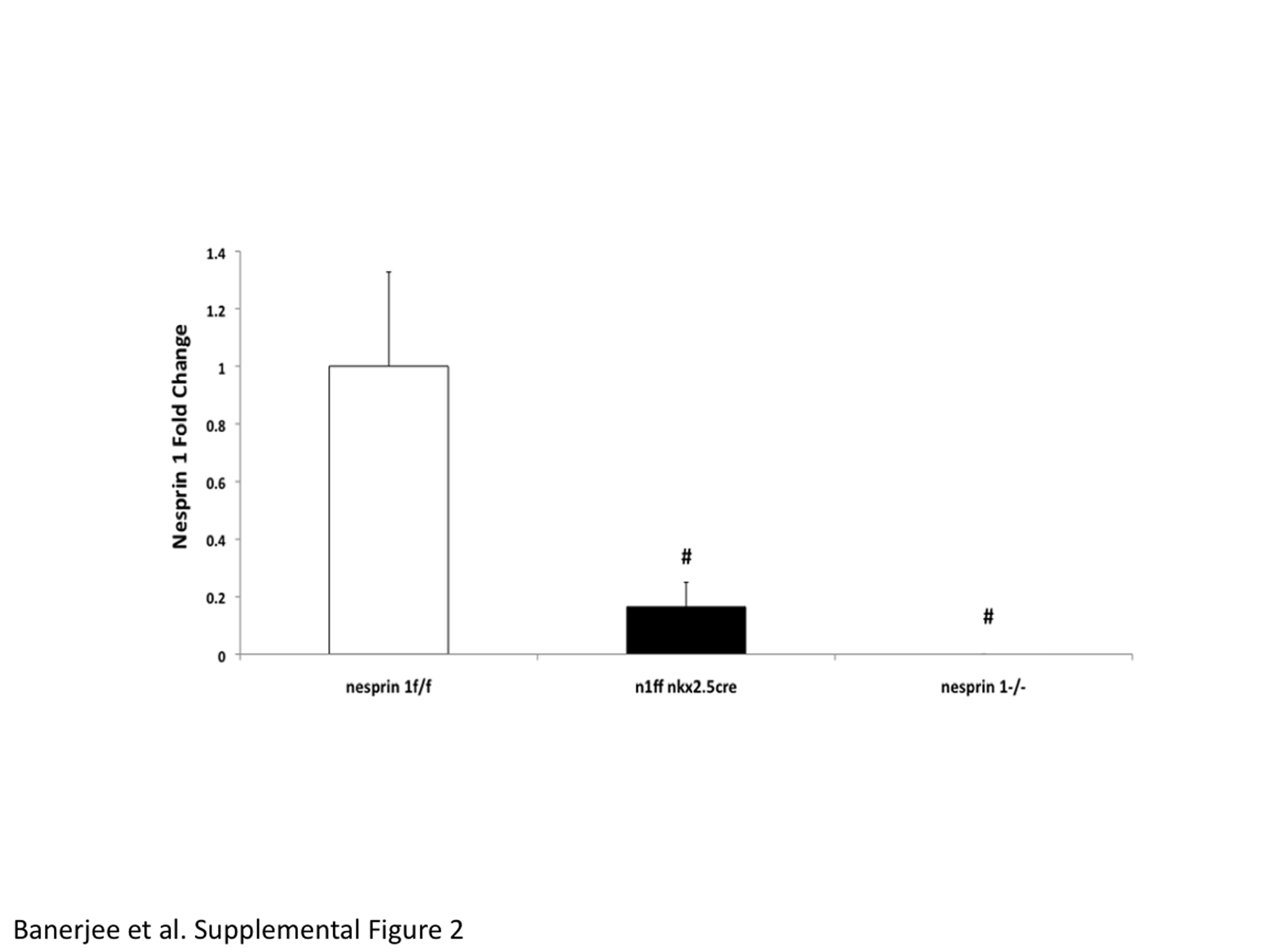

Supplement: Figure S2 — Validation of knockout of Nesprin 1 in cardiac specific deficient mice. Nesprin 1 Real Time PCR analyses of isolated cardiomyocytes from Wild Type, nesprin1f/f;Nkx2.5Cre and global nesprin1−/− mice. We observed an ∼88% decrease observed nesprin1f/f;Nkx2.5Cre isolated cardiomyocytes. student-t test #P<0.01. (TIF) [file pgen.1004114.s002.tif]

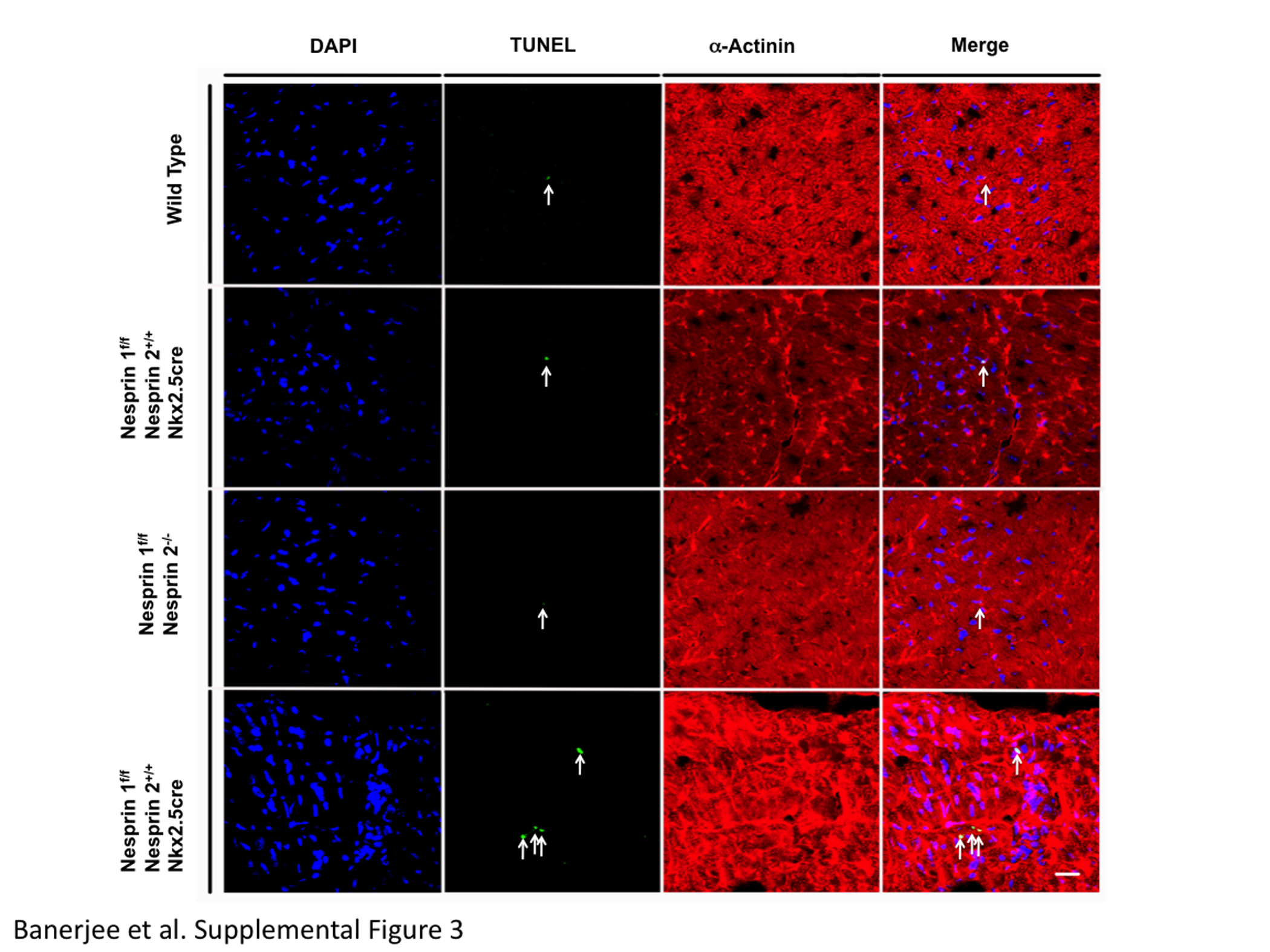

Supplement: Figure S3 — Representative TUNEL stain in Nesprin 1 and/or 2 knockout hearts. Blue = DAPI, Green = TUNEL, Red = α-actinin White arrows indicate apoptotic nuclei. (TIF) [file pgen.1004114.s003.tif]

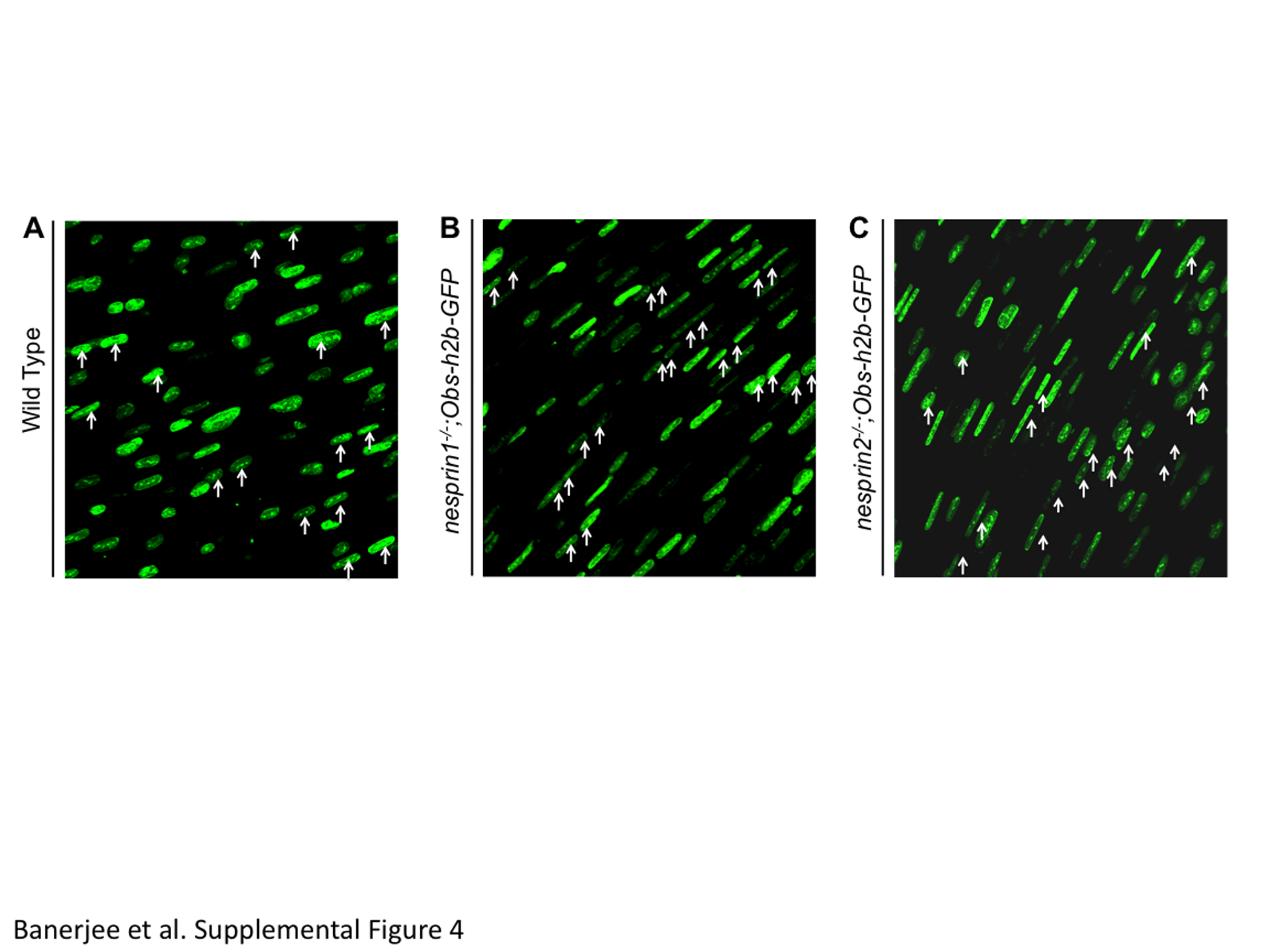

Supplement: Figure S4 — Analyses of Nesprin 1 and Nesprin 2 knockout hearts. Representative 3D reconstruction, 30 µm thick images from (A) Wild Type;Obs-H2bGFP (B) nesprin1−/−;Obs-h2b-GFP and (C) nesprin2−/−;Obs-h2b-GFP Hearts. White arrows indicate cardiomyocyte nuclei. #p<0.01. (TIF) [file pgen.1004114.s004.tif]

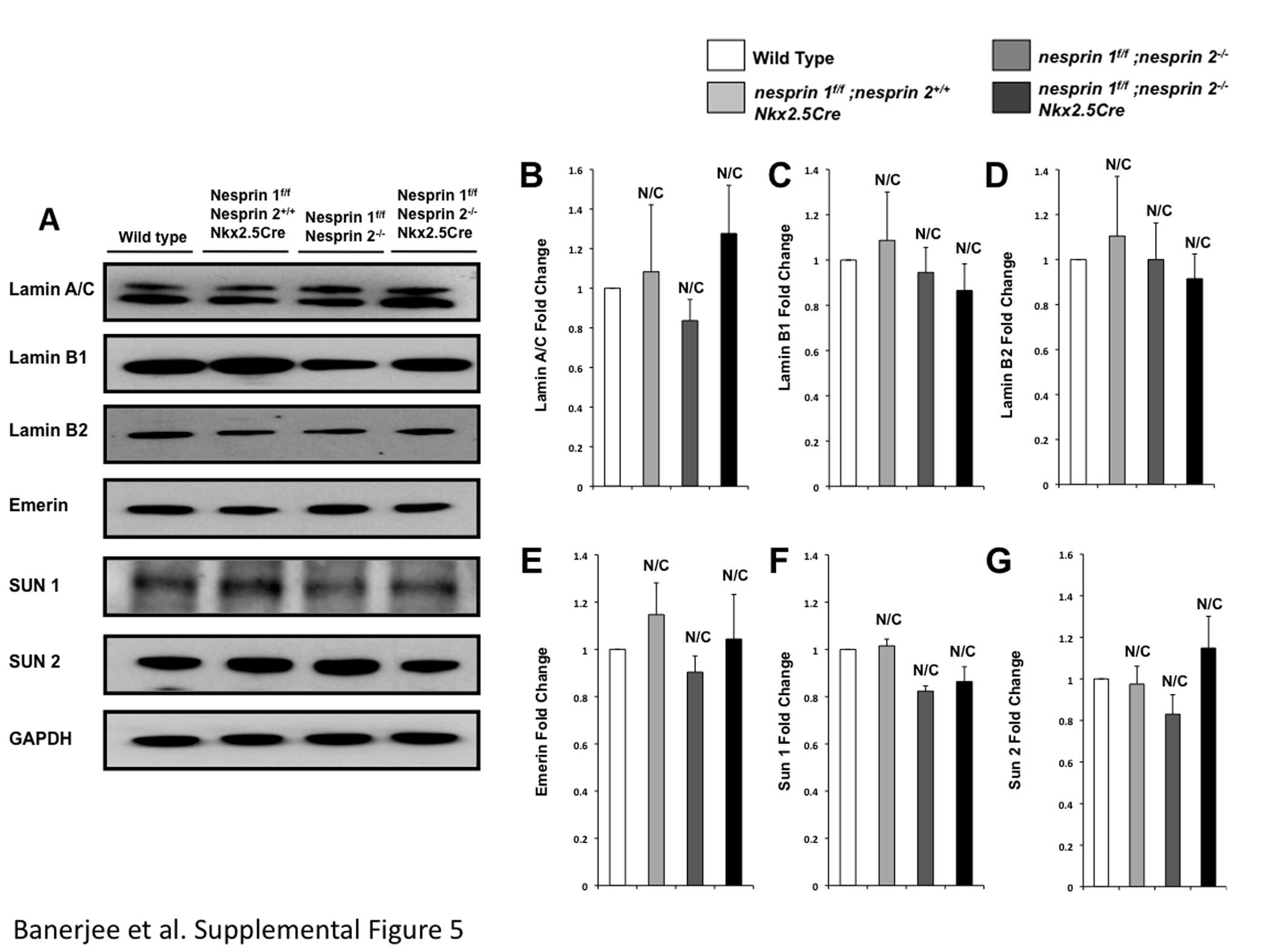

Supplement: Figure S5 — Western LINC complex and LINC complex associated factors. (A) Representative western blots for Lamin A/C, Lamin B1, Lamin B2, Emerin, SUN1, SUN2 and GAPDH. Western samples are representative of N = 3 cardiomyocyte isolations, (B–G) Quantification of Western analyses for LINC Complex and LINC Complex associated factors. ANOVA with a post hoc Bonferroni test N/C = No Change. (TIF) [file pgen.1004114.s005.tif]

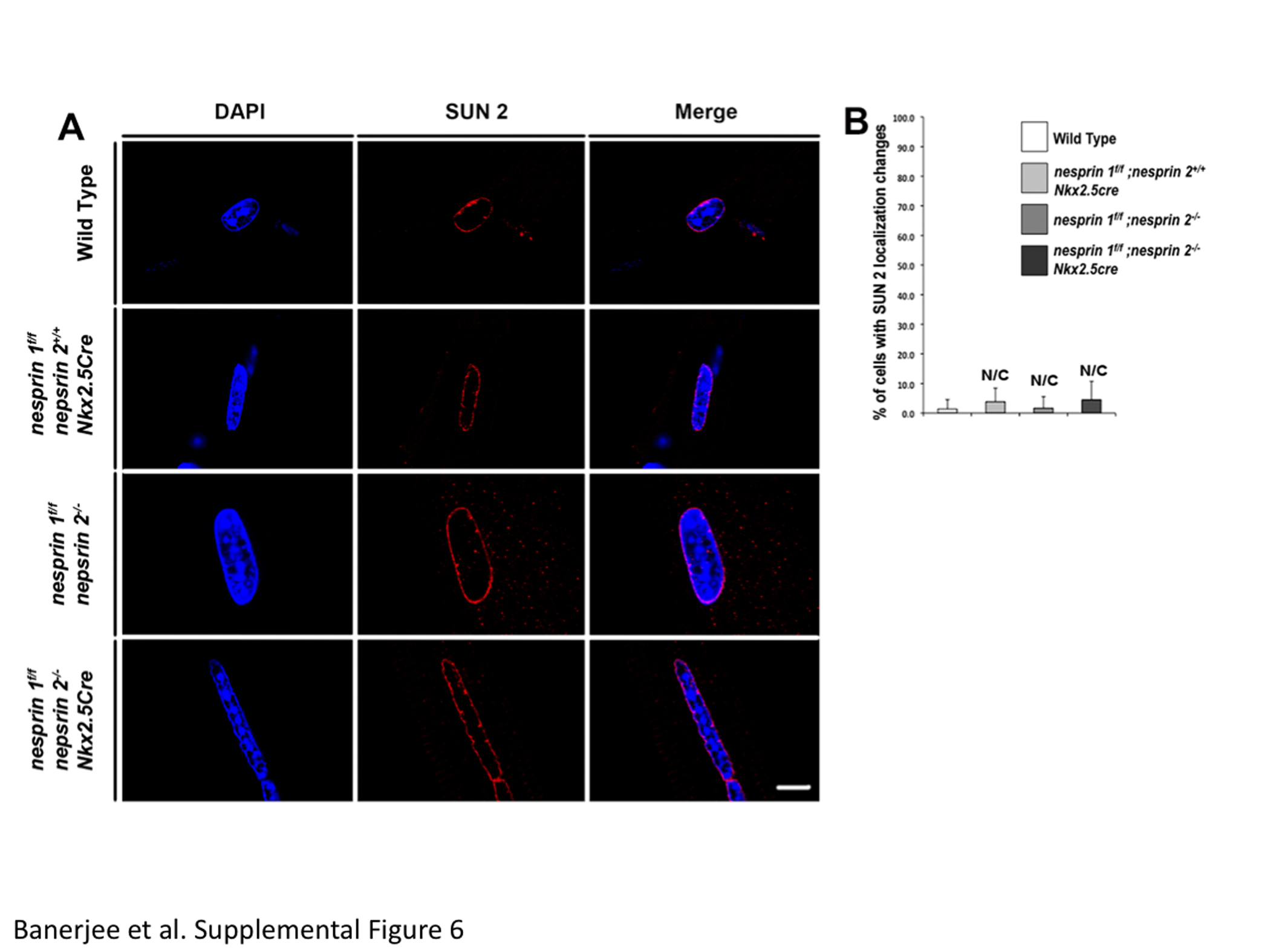

Supplement: Figure S6 — Loss of Nesprin 1 and 2 does not cause abnormal SUN 2 staining. (A) Representative immunofluorescence nuclear images from isolated adult cardiomyocytes (B) Quantification of percent cells with abnormal SUN 2 staining. Blue = DAPI, Red = SUN 2 ANOVA with a post hoc Bonferroni test #p<0.01. White Bar = 7 µm. (TIF) [file pgen.1004114.s006.tif]

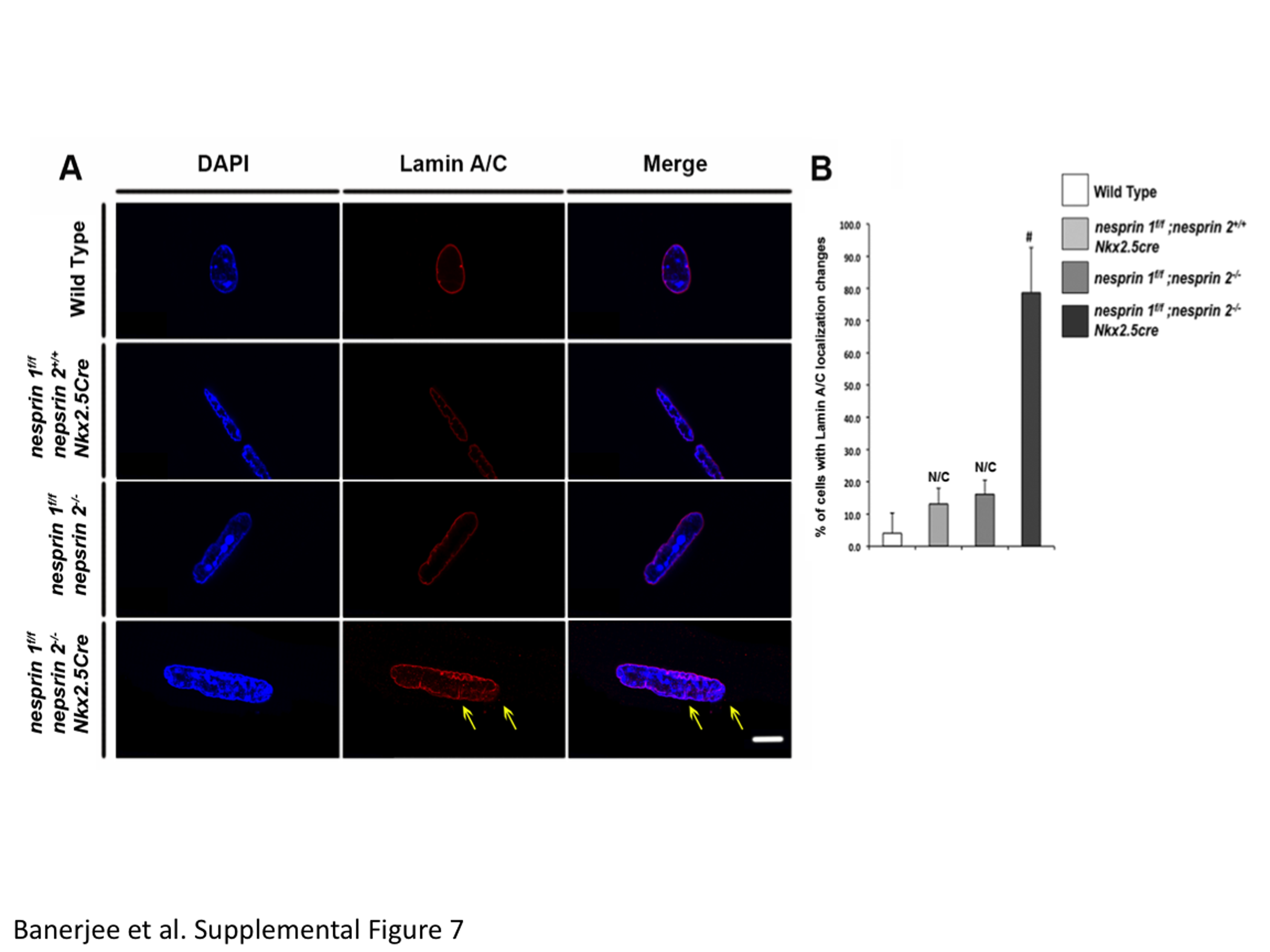

Supplement: Figure S7 — Loss of both Nesprin 1 and 2 had a significant impact on the localization patterns of Lamin A/C. (A) Representative immunofluorescence nuclear images from isolated adult cardiomyocytes (B) Quantification of percent cells with abnormal Lamin A/C staining. Blue = DAPI, Red = Lamin A/C ANOVA with a post hoc Bonferroni test #p<0.01. White Bar = 7 µm Yellow arrows = Abnormal Lamin A/C Stain. (TIF) [file pgen.1004114.s007.tif]

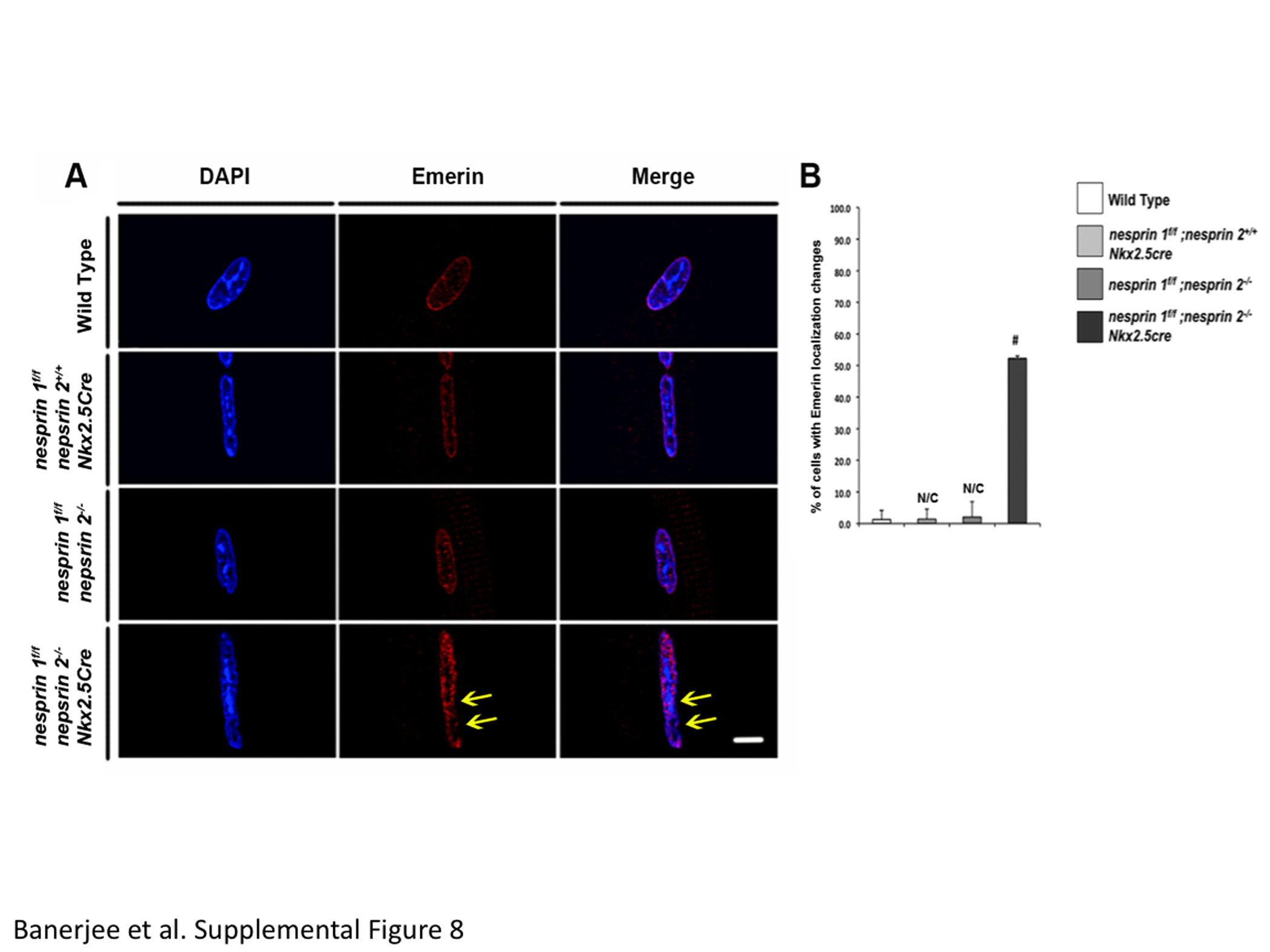

Supplement: Figure S8 — Loss of both Nesprin 1 and 2 had a significant impact on the localization patterns of Emerin. (A) Representative immunofluorescence nuclear images from isolated adult cardiomyocytes (B) Quantification of percent cells with abnormal Emerin staining. Blue = DAPI, Red = Emerin ANOVA with a post hoc Bonferroni test #p<0.01. White Bar = 7 µm, Yellow arrows = Abnormal Emerin Stain. (TIF) [file pgen.1004114.s008.tif]

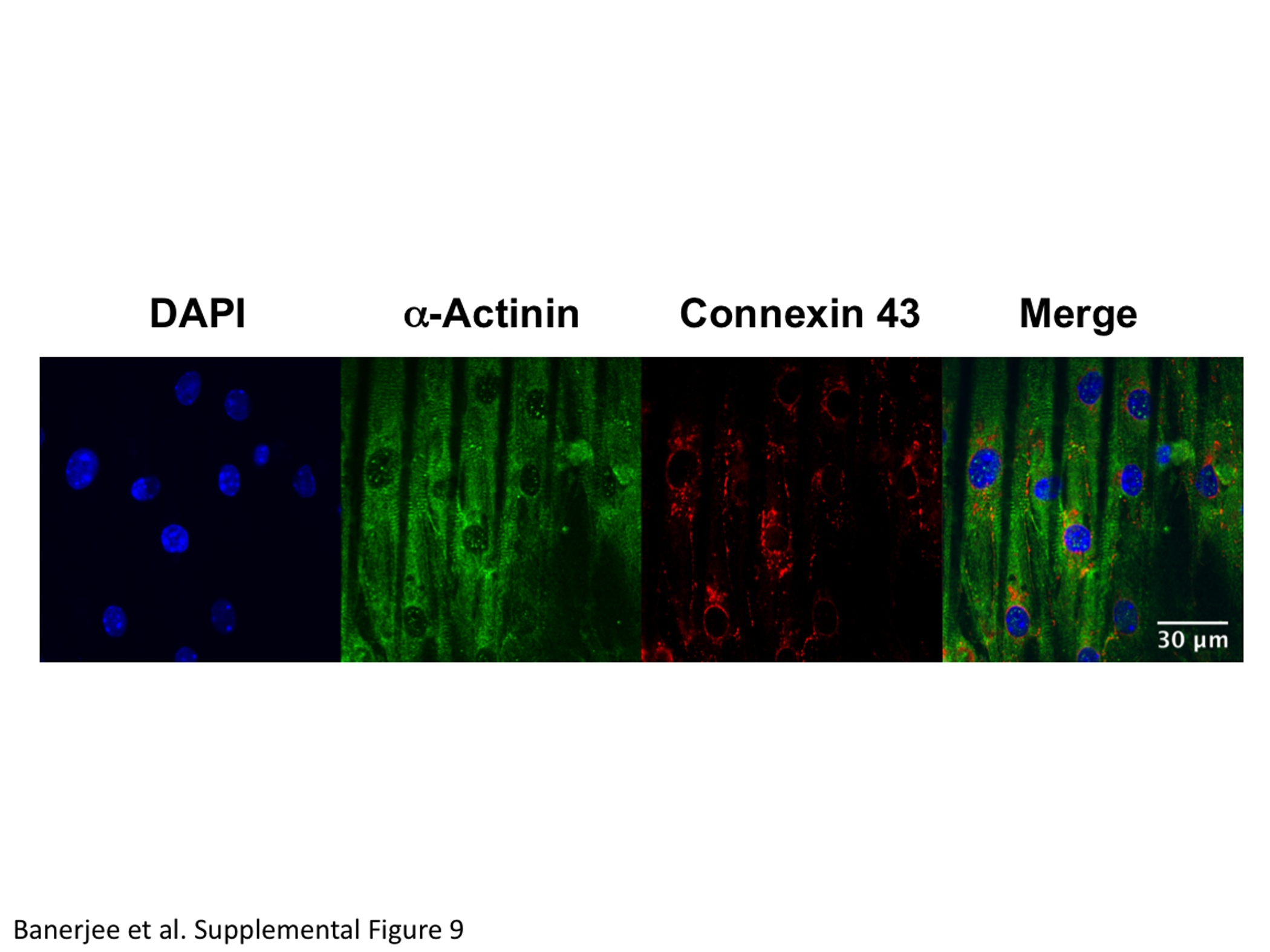

Supplement: Figure S9 — Representative image of isolated cardiomyocytes on aligned, micropatterned substrates. Representative image of day 1–2 neonatal cardiomyocytes on aligned substrates. Cardiomyocytes appear rod-like and form cell-cell connections. (Green = α-actinin, Blue = DAPI, Red = Connexin-43). (TIF) [file pgen.1004114.s009.tif]
