# Supplementary material for: Targeted Ablation of Nesprin 1 and Nesprin 2 from Murine Myocardium Results in Cardiomyopathy, Altered Nuclear Morphology and Inhibition of the Biomechanical Gene Response
Source: PLoS Genet. 2014 Feb 20;10(2):e1004114. doi: 10.1371/journal.pgen.1004114 (PMC3930490; doi:10.1371/journal.pgen.1004114)
Supplement: Table S1 — Real Time PCR Primers for study. (DOCX) [file pgen.1004114.s010.docx]

| **Genotyping Primers** |  |  |  |
| --- | --- | --- | --- |
| Nesprin 2 GP1 | 5'-AGTGCTTCCTAAGGCTAGTCCAG-3' | | |
| Nesprin 2 GP3 | 5'-GCAGCAGCTCAAGTCTGACATCGGC-3' | | |
| Nesprin 2 GP4 | 5'-TCTACAGTCCAGATCTTTCCACCCC-3' | | |
| **Semi Quantitative Real Time PCR Primers** | |  |  |
| N2S WF | 5'-GCAGCAGCTCAAGTCTGACATCGGC-3' | | |
| N2S R | 5'-CTGCGGCTCTCTGCTGTCGCTAGC-3' | | |
| **Real Time PCR Primers** |  |  |  |
| Nesprin 1 f | 5'-CAGTACAAGCTCAGCAGAGTGAA-3' | | |
| Nesprin 1 r | 5'-GCTCATTCAGTTTCCTCTGTATTTC-3' | | |
| Nesprin 2 f | 5'-CGAGCTGGAAGCTCTGAAGT-3' | | |
| Nesprin 2 r | 5'-ATGGAGTCTATTTTGGAGTTCTGTG-3' | | |
| BNP f | 5'-TGTTTCTGCTTTTCCTTTATCTGTC-3' | | |
| BNP r | 5'-CTCCGACTTTTCTCTTATCAGCTC-3' | | |
| Egr-1 f | 5'-CCTATGAGCACCTGACCACA-3' | | |
| Egr-1 r | 5'-TCGTTTGGCTGGGATAACTC-3' | | |
| Iex-1 F | 5'-TTATAGGGTCGGTAAGACAGAGTTG-3' | | |
| Iex-1 r | 5'-GACGGAGTGTTACCCCTAATCTTAT-3' | | |
| c-fos f | 5'-AGCCCCTGTGTACTCCCGTG-3' | | |
| c-fos r | 5'-GCCTTGCCTTCTCTGACTGC-3' | | |
| c-jun f | 5'-TTCCTCCAGTCCGAGAGCG-3' | | |
| c-jun r | 5'-TGAGAAGGTCCGAGTTCTTGG-3' | | |
| c-myc f | 5'-ATGCCCCTCAACGTGAACTTC-3' | | |
| c-myc r | 5'-GTCGCAGATGAAATAGGGCTG-3' | | |

Banerjee et al. Supplemental Table 1
